# Rapid digital pathology of H&E-stained fresh human brain specimens as an alternative to frozen biopsy

**DOI:** 10.1038/s43856-023-00305-w

**Published:** 2023-05-30

**Authors:** Bhaskar Jyoti Borah, Yao-Chen Tseng, Kuo-Chuan Wang, Huan-Chih Wang, Hsin-Yi Huang, Koping Chang, Jhih Rong Lin, Yi-Hua Liao, Chi-Kuang Sun

**Affiliations:** 1 Department of Electrical Engineering and Graduate Institute of Photonics and Optoelectronics, National Taiwan University, Taipei, Taiwan; 2 Division of Neurosurgery, Department of Surgery, National Taiwan University Hospital, Taipei, Taiwan; 3 Department and Graduate Institute of Pathology, National Taiwan University Hospital, Taipei, Taiwan; 4 Department of Dermatology, National Taiwan University Hospital and National Taiwan University College of Medicine, Taipei, Taiwan; 5 Graduate Institute of Biomedical Electronics and Bioinformatics, National Taiwan University, Taipei, Taiwan; 6 Molecular Imaging Center, National Taiwan University, Taipei, Taiwan

**Keywords:** CNS cancer, Brain imaging, Pathology, Prognostic markers

## Abstract

**Background:**

Hematoxylin and Eosin (H&E)-based frozen section (FS) pathology is presently the global standard for intraoperative tumor assessment (ITA). Preparation of frozen section is labor intensive, which might consume up-to 30 minutes, and is susceptible to freezing artifacts. An FS-alternative technique is thus necessary, which is sectioning-free, artifact-free, fast, accurate, and reliably deployable without machine learning and/or additional interpretation training.

**Methods:**

We develop a training-free true-H&E Rapid Fresh digital-Pathology (the-RFP) technique which is 4 times faster than the conventional preparation of frozen sections. The-RFP is assisted by a mesoscale Nonlinear Optical Gigascope (mNLOG) platform with a streamlined rapid artifact-compensated 2D large-field mosaic-stitching (rac2D-LMS) approach. A sub-6-minute True-H&E Rapid whole-mount-Soft-Tissue Staining (the-RSTS) protocol is introduced for soft/frangible fresh brain specimens. The mNLOG platform utilizes third harmonic generation (THG) and two-photon excitation fluorescence (TPEF) signals from H and E dyes, respectively, to yield the-RFP images.

**Results:**

We demonstrate the-RFP technique on fresh excised human brain specimens. The-RFP enables optically-sectioned high-resolution 2D scanning and digital display of a 1 cm^2^ area in <120 seconds with 3.6 Gigapixels at a sustained effective throughput of >700 M bits/sec, with zero post-acquisition data/image processing. Training-free blind tests considering 50 normal and tumor-specific brain specimens obtained from 8 participants reveal 100% match to the respective formalin-fixed paraffin-embedded (FFPE)-biopsy outcomes.

**Conclusions:**

We provide a digital ITA solution: the-RFP, which is potentially a fast and reliable alternative to FS-pathology. With H&E-compatibility, the-RFP eliminates color- and morphology-specific additional interpretation training for a pathologist, and the-RFP-assessed specimen can reliably undergo FFPE-biopsy confirmation.

## Plain language summary

Brain tumors can be fatal and surgery is often required to remove them. During surgery, clinicians need to look for any leftover tumor tissue so that recurrence of the disease can be avoided. This requires sectioning of frozen tissue samples, staining them, and visualizing structural details under a microscope in the lab. This process should be fast to make the operation shorter and safer for the patient. Here, we provide an alternative approach to staining and imaging tumor samples, which is much faster than the current process. We show that our approach works with fresh tumor samples, avoiding the need to freeze and physically section them. We can distinguish normal versus tumor tissues, and pathologists do not require special training to use our approach. Our approach might ultimately help to improve the speed, safety, and outcomes of brain tumor surgery.

## Abstract (web summary)

Borah et al. develop a rapid digital pathology approach for the analysis of fresh tissue specimens, avoiding the need for frozen section histopathology. They apply the method to fresh human brain specimens and demonstrate their ability to distinguish normal and tumor regions.

## Introduction

Although an FFPE pathology provides ultimate reliability, it is not suitable for ITA as it takes up-to 1–2 days of processing time. With a shorter assessment time, FS-pathology is presently the global standard for an ITA. However, FS involves cryosectioning, thus becomes labor intensive, and a single round consumes up-to 30 min of duration, eventually limiting feasible rounds of ITAs. Furthermore, FS-biopsy is susceptible to several artifacts which might compromise an assessment^1–4^. There is thus an apparent necessity for an FS-alternative technique which is sectioning-free, artifact-free, portable, fast, accurate, applicable to fresh specimens, and most importantly, based on the global gold standard H&E dyes to make it reliably deployable without machine learning and/or additional interpretation training for a pathologist.

In the paradigm of modern-era computer-aided digital pathology^5–7^, whole slide imaging (WSI), and stitched panoramic virtual slides (VSs) are rapidly evolving, which helps archive billions of FS/FFPE histopathology slides in digital form to assist with any-time re-assessment, and further enables a pathologist to perform rapid remote assessments with easy WSI-VS access from any location. However, to digitally preserve decent diagnostic reliability, it is crucial that there is no loss of resolution amidst the pixel pathway from the specimen to the digital display system. According to the practical guidelines from the Digital Pathology Association^8^, a WSI VS with a typical ×20 magnification is usually acceptable for standard viewing and interpretation, while a base ×40 with a 0.5 µm digital resolution with a Nyquist- satisfied^9,10^ 0.25 µm pixel size at 24-bit color depth is often expected to be maintained. That requires a 1 × 1 mm^2^ area to comprise ≥384 M bits, which would extend to ≥38.4 G bits or 1.6 Gigapixels for a 1 cm^2^ area. For such ×40 or even ×20 objective lens with a high numerical aperture (NA, close to or greater than 1), the system field-of-view (FOV) is often limited to <1 mm^2^, which mandates a series of stitching operations, which are expected to be artifact-free to not compromise the diagnostic reliability. Nevertheless, despite utilizing high-speed brightfield CCD/CMOS sensors, the prevailing WSI solutions^11–14^ often required up-to a minute or more for scanning and/or rendering post-acquisition computations for multi-gigapixel centimeter-scale acquisition, stitching, and display operations, especially when considering a high-magnification high-NA objective lens.

In the context of surgical pathology ITA, rapid evaluation is a primary requirement to minimize the net surgery time to keep the patient safe. Unlike physical sectioning involved in FS/FFPE, several ITA-capable digital imaging modalities were reported with optical virtual sectioning that enabled faster assessments. For a fresh unfixed unsectioned tissue, it is expected to digitize as much information as possible during the ITA itself, so that decent diagnostic reliability is digitally secured, and the tissue does not require to be fixed or deep-frozen and physically stored for any re-assessment. However, being targeted to a digital surgical pathology ITA, the prior arts^15–28^ as enlisted in Table 1 either might not reach the Nyquist-satisfied gigapixel-sampled half-a-micron digital resolution to meet the state-of-the-art WSI standard^8,11,14^, and/or might not enable a real-time large-field artifact-free stitching/mosaicking feature and/or a subminute gigapixel acquisition and digital display ability with an uncompromised resolution across the specimen-to-digital-display pixel pathway. Besides, while utilizing alternatives to the gold standard H&E staining, specific machine learning algorithms^25,26,28^ or additional interpretation training^15–21,24,29^ would be required, because the pathologists are often trained and adapted to histological features expressed with typical H&E characteristic appearance. For instance, while utilizing nuclei-staining dyes^20,30–32^ alternative to hematoxylin, the associated chemical interaction might not precisely match an H&E interaction. Therefore, in such a case, it is often required to investigate or validate the staining outcomes. Furthermore, for different organs, the staining outcomes might be different, eventually requiring repeated investigations/validations and additional training. Hematoxylin-compatibility^33–35^ thus becomes quite helpful for a training-free yet reliable FS/FFPE alternative, which demands a nonlinear multi-harmonic generation approach owing to the lack of fluorescence from hematoxylin dyes^36^.

**Table 1:** A comparison of state-of-the-art technologies applicable in intraoperative tumor assessment.

Tech.	Ref.	FOV (mm × mm) (approx.)	Pixel number or pixel size	Accuracy	Sensitivity	Specificity	H&E compatibility	Real-time^a^ stitching and half-a-micron resolution display	Tissue
CM	^15^	0.37 × 0.37	–	–	95%	100%	No	No	Brain
	^16^	–	1024 × 1024	94.4%	89.7%	95.3%	No	No	Skin
	^17^	–	1024 × 1024	–	76%–90%	85%–98%	No	No	Prostate
OCT	^18^	5.0 (diameter)	–	–	88.9–92.6%	96.8–98.4%	No	No	Skin
MUSE	^19^	4.0 (diameter)	2748 × 2200	96.03%	97.62%	92.86%	No	No	Breast
MPM	^20^	0.48 × 0.48	1024 × 1024	94.1%	95.4%	93.3%	No	No	Breast
	^21^	1.0 × 1.0	2048 × 2048	98.3%	97.3%	100%	No	No	Prostate
SIM	^22^	1.3 × 1.3	2048 × 2048	76.5%–82.4%	62.5%–87.5%	77.8%–88.9%	No	No	Prostate
	^23^	–	–	89.2%	79.2%	95.1%	No	No	Kidney
LSM	^24^	0.9 (width)	0.9 µm/pixel	93%	90%	94%	No	No	Prostate
SRS	^25^	0.4 × 0.4	1024 × 1024	>92%	94.5%	94.1%	No	No	Brain
	^26^	0.4 × 0.4		92%–96%	–	–	No	No	Brain
	^27^	–	–	87%	–	–	No	No	Skull
UV-PAM	^28^	0.5 × 0.5	700 × 500	91.7–96.9%	91.3–96.5%	91.5–96.9%	No	No	Colon and liver
FS	^4^	–	–	96.96%	97.23%	96.30%	Yes	–	–
FFPE (global)		–	–	100%	100%	100%	Yes	–	–
the-RFP (this report)		≥1.0 × 1.0	6000 × 6000	100%	100%	100%	Yes	Yes	Brain

The numbers are based on prior reports and to the best of the author’s knowledge.

*CM* confocal microscopy, *OCT* optical coherence tomography, *MUSE* microscopy with ultraviolet surface excitation, *MPM* multiphoton optical microscopy, *SIM* structured illumination microscopy, *LSM* light sheet microscopy, *SRS* stimulated Raman scattering, *UV-PAM* ultraviolet photoacoustic microscopy, *FS* frozen section, *FFPE* formalin-fixed paraffin-embedded, *the-RFP* true-H&E Rapid Fresh digital-Pathology.

^a^Mosaicking/stitching is considered real-time when stitching operations are performed parallelly with the multi-tile acquisition, and there is no drop in the overall frame rate or no considerable waiting time owing to undergoing the stitching-specific computations. Clinical practice of non-H&E methods would require specific machine learning algorithms^25,26,28^ or additional interpretation training^15–21,24,29^.

Here we report an FS-alternative ITA-capable technique called Training-Free True-H&E Rapid Fresh digital-Pathology (the-RFP) that uses physical-sectioning-free whole-mount specimens rapidly stained with the gold standard H&E dyes, with no additional interpretation training to a pathologist. The-RFP is assisted by a mesoscale Nonlinear Optical Gigascope (mNLOG) with a high Nyquist-figure-of-merit (NFOM)^37^. In the domain of digital surgical pathology, our mNLOG platform, for the first time to the best of our knowledge, provides a truly WSI-competing whole specimen superficial imaging (WSSI) digital ITA solution enabling multicolor imaging of a 1 cm^2^ area in <120 s with a total of 86 G bits or 3.6 Gigapixels (24-bit) preserving a submicron digital resolution with no requirement of post-acquisition data/image processing. It is noted that despite substantial contributions being made towards image/video or panoramic stitching techniques^38–43^, feature-based sophisticated algorithms are often not suitable for a large-field high-pixel-rate dynamic microscopy in the context of parallel implementation, distortion compensation, immunity to high-frequency noise, and especially to assist with half-a-second computational complexity for real-time stitching of ultra-high resolution (such as >800 M bit) imaging tiles. To fit our specific need, a Compute Unified Device Architecture (CUDA)-accelerated rapid artifact-compensated 2D large-field mosaic-stitching (rac2D-LMS) approach is streamlined to our large-FOV (≥1 mm^2^) high-NFOM (>1) multi-channel nonlinear optical laser-scanning and acquisition system. rac2D-LMS involves an instant radial distortion compensation based on a simplified FOV-distortion model^44^, followed by a direct pixel and position-based spatial cross-correlation with an ability to render mosaic-stitching of >12 × 12 mm^2^ area with 130 G bits in 60 s.

To demonstrate the-RFP, excised fresh human brain specimens were utilized for rapid histopathological assessment of tumor-specific features. Note that despite unprecedented revolutions in medical sciences and technologies, the 5-year survival for glioblastoma cases only improved from 4% to 7% within the period of 1975–1977 to 2009–2015^45^. Therefore, it is critical to enable a fast and reliable ITA to help effectively resect such a fatal tumor while mostly preserving healthy adjacent regions to help circumvent post-operative complications. A sub-6-minute True-H&E Rapid whole-mount-Soft-Tissue Staining (the-RSTS) protocol is introduced, which is dedicated to soft and frangible unfixed human brain specimens. The-RFP combines THG and TPEF signals from the traditional H and E dyes, respectively. Considering specimen-to-digital-display cumulative assessment time of <8 min, the-RFP provides a 4× faster assessment than a typical FS-biopsy while revealing a dramatic improvement compared to the prior high-resolution human brain ITA-potential studies (>1 h/cm^2^)^46–48^ in the contexts of both speed and reliability.

The streamlined specimen-to-digital-display service of the-RFP assisted by mNLOG platform offers an excellent throughput solution, effectively sustained at >700 M bits/sec, for a rapid digital ITA, where the perpetual topical whole-mount H&E staining approach the-RSTS allows physical-sectioning-free rapid pathology readings with the traditional H&E specific histopathological features. To investigate the-RFP reliability, a non-interventional non-inferiority blind clinical study was conducted. With zero additional interpretation training for our collaborating pathologists, blind tests reveal an excellent accuracy of 100% considering a total of 50 normal and tumor-specific human brain specimens (obtained from 8 participants), where the-RFP-based decisions matched the respective decisions from FFPE-biopsies.

## Methods

### The clinical study

All experiments were performed as per the protocol reviewed and approved by the Research Ethics Committee of National Taiwan University and National Taiwan University Hospital. Tumor-specific specimens were collected from 4 participants (diffuse gliomas of WHO grade 4) undergoing tumor resection surgeries. We collaborated with the Division of Neurosurgery, Department of Surgery, and Department and Graduate Institute of Pathology of National Taiwan University Hospital. Institutional Review Board (IRB) approval was taken under a project entitled “Microscopy Imaging of Hematoxylin-Eosin Stained Human Brain Tissues: Assessment margins of Excised Glioma Tumor in Surgery,” project number: 201912225RINB (National Taiwan University Hospital). Informed consent from each patient was ensured. Additionally, confirmed normal brain specimens were collected from preserved brain tissues obtained from 4 participants, under an IRB-approval entitled “Developing immunoassays for early detection of Alzheimer’s disease,” project number: 201412063RINC (National Taiwan University). Tumor-specific tissues were collected based on disease coverage and tissue availability from the Division of Neurosurgery, Department of Surgery, National Taiwan University Hospital; choices were random otherwise. Sex and gender were not relevant to this study.

### Resonant-galvo large-angle raster scanning system

The mNLOG platform reported in this study is powered by a Large Angle Optical Raster Scanning (LAORS) system^37,49^. For nonlinear optical excitation, we employed a 70 MHz pulsed laser source (Fidelity-2 Yb-fiber laser, Coherent, Inc., USA) with a central wavelength of 1070 nm. A high repetition rate, in this case, helps secure a high NFOM that enables an optical-zoom-free high digital resolution. It is noted that this study combines THG and TPEF for the detection of hematoxylin and eosin signals, respectively. A 4 kHz resonant scanner (CRS 4 kHz, Cambridge Technology, USA) was used for fast (horizontal X)-axis scanning, while a slower galvanometric scanner (8320 K, Cambridge Technology, USA) was employed along the vertical (Y) axis to accomplish a fast resonant-galvo laser raster scanning. The optical relay system comprises our proprietary tube lens with an effective focal length (EFL) of 167 mm, together with an off-the-shelf scan lens (LSM05-BB, Thorlabs, USA) with an EFL of 110 mm. For focusing the scanning beam over a tissue sample, a high NA (0.95) ×20 objective lens was employed (XLUMPlanFl, ×20/0.95 W, Olympus). For efficient signal collection, a second relay system with a demagnification factor of 3.75 was employed for each channel. Each relay system comprises two focusing lenses to be denoted as L0 and L1-2 with EFLs of 150 mm and 40 mm, respectively. A long-pass beam splitter FF735-Di02, Semrock, was utilized as a primary dichroic (D1). A second dichroic (D2, T505lpxr, Chroma) reflects THG signal to a photomultiplier tube (PMT 1), and transmits TPEF signal to PMT 2. It is noted that the proposed mNLOG imaging platform is capable of providing 3-channel RGB tiles and can perform 3-channel automatic mosaic-stitching. To implicate the-RFP technique, we made use of 2 channels for the detection of THG and TPEF signals. Appropriate band-pass filters (F1, 355/10, Edmund Optics, and F2, 575/50, Edmund Optics) were utilized to ensure the detection of THG and TPEF signals. A color glass filter (FGB37-A, Thorlabs) was added in series with each band pass filter.

For the-RFP operation demonstrated in this study, an imaging tile was set to an FOV of 1 mm^2^ comprising 6000 × 6000 pixels, ensuring a pixel size of 167 nm. For each specimen, multiple tiles were acquired with the proposed mNLOG platform to provide a large cumulative viewing area. In each case, a sustained effective throughput of >700 M bits/sec was secured, including all time-penalties across the specimen-to-digital-display pixel pathway. It is noted that the number of slow-axis-lines was set to 6000 in order to exceed the limiting condition of the Nyquist-Shannon criterion considering a theoretical resolution of 429 nm (for 2-photon process, 1070 nm, 0.95 NA). See our previous publication^37^ for a detailed point spread function (PSF) analysis. Nevertheless, it is feasible to lower the number of slow-axis-lines so as to enlarge the pixel-size up-to the limiting Nyquist-requirement, which would further boost the cumulative imaging speed.

### Multi-channel data acquisition

A state-of-the-art digitizer, ATS9440 (Alazar Technologies Inc., Canada), was employed to support up-to 125 Mega Samples per second (MSps) of sampling rate for up-to 4 simultaneously digitizable channels. Limited by our laser repetition rate of 70 MHz, we synchronized each sampling event to each laser pulse enabling a 70 M/s sampling rate. The pulse-sync signal output from Fidelity-2 was fed to the external clock input of ATS9440 to enable a synchronized acquisition. It is noted that each of our imaging tiles comprised 6000 pixels along each fast-axis line. Therefore, an effective 1–2 optical pulse(s) per pixel was ensured. For slow axis synchronization with the fast axis, a multifunction I/O card, PCIe-6341 (National Instruments Corporation, USA), was employed, providing a sawtooth waveform to the galvanometer scanning mirror driver. Note that following a straightforward idea^50^, nonlinear distortion caused by the cosinusoidal motion of the resonant scanning mirror was corrected in real-time prior to applying rac2D-LMS. For current-to-voltage conversion of the PMT output signal, a wide-bandwidth transimpedance amplifier C6438-01 (Hamamatsu Photonics, Japan) was employed for each channel, to be denoted as TA1 and TA2. The voltage outputs from C6438-01 units were parallelly digitized by ATS9440. To laterally displace a specimen for centimeter-scale imaging, we employed two motorized linear stages along the horizontal and vertical axes (TSDM40-15X, Sigma Koki, Japan). Each stage provides a maximum lateral moving speed of around 5.6 mm/s. While collecting multiple tiles, an overlapping of around 9% of the FOV-width or -height was ensured. It is noted that CUDA (v11.4)-accelerated OpenCV (C++) v4.5.2 was utilized for streamlined data/image processing powered by a Quadro RTX 8000 (NVIDIA Corporation, USA) graphics card. A C++-based software LASERaster^+^ (v4.1.0.4)^49^ was employed for data acquisition and processing. For a detailed illustration of the idea and construction of a high-NFOM imaging system, see our previously published papers^37,49^.

### Instantaneous distortion compensation model in rac2D-LMS

Assuming an input image I_*D*_ (*x, y*) with C×R pixels, we first define two 2D arrays as follows

1 $${{{{{{\rm{F}}}}}}}_{x}(x,y)={\left(\begin{array}{ccc}0 & \cdots & {{{{{\rm{C}}}}}}-1\\ \vdots & \ddots & \vdots \\ 0 & \cdots & {{{{{\rm{C}}}}}}-1\end{array}\right)}_{{{{{{\rm{C}}}}}}\times {{{{{\rm{R}}}}}}}\,{{{{{\rm{and}}}}}}$$

2 $${{{{{{\rm{F}}}}}}}_{y}(x,y)={\left(\begin{array}{ccc}0 & \cdots & 0\\ \vdots & \ddots & \vdots \\ {{{{{\rm{R}}}}}}-1 & \cdots & {{{{{\rm{R}}}}}}-1\end{array}\right)}_{{{{{{\rm{C}}}}}}\times {{{{{\rm{R}}}}}}}.$$

Defining two spatial scaling parameters ${{{{{{\rm{S}}}}}}}_{x}$ and ${{{{{{\rm{S}}}}}}}_{y}$, and following an FOV-distortion model^44^, we evaluate

3 $${r}_{d}(x,y)=\frac{360}{{{{{{\rm{A}}}}}}\pi }\,{\tan }^{-1}\left[2{r}_{u}(x,y)\tan \frac{{{{{{\rm{A}}}}}}\pi }{720}\right],$$

$${{{{{\rm{where}}}}}}\,{r}_{u}(x,y)=\sqrt{{r}_{x}{(x,y)}^{2}+{r}_{y}{(x,y)}^{2}}\,{{{{{\rm{and}}}}}}$$

$${r}_{x}(x,y)=\frac{1}{{c}_{1}}({{{{{{\rm{F}}}}}}}_{x}(x,y)-{c}_{1}),\,{r}_{y}(x,y)=\frac{1}{{c}_{2}}({{{{{{\rm{F}}}}}}}_{y}(x,y)-{c}_{2}),$$

$${c}_{1}=0.5\,{{{{{\rm{C}}}}}}(1.0+{{{{{{\rm{X}}}}}}}_{off}/{{{{{{\rm{S}}}}}}}_{x}),\,{c}_{2}=0.5\,{{{{{\rm{R}}}}}}(1.0+{{{{{{\rm{Y}}}}}}}_{off}/{{{{{{\rm{S}}}}}}}_{y}).$$

A multiplicative factor $f(x,y)$ is obtained as ${r}_{u}(x,y)/{r}_{d}(x,y)$ in the case of pincushion distortion, or as ${r}_{d}(x,y)/{r}_{u}(x,y)$ in the case of barrel distortion. Two pixel-coordinate maps ${{{{{{\rm{M}}}}}}}_{x}(x,y)$ and ${{{{{{\rm{M}}}}}}}_{y}(x,y)$ for the horizontal and vertical axes, respectively, are obtained as

4 $${{{{{{\rm{M}}}}}}}_{x}(x,y)={c}_{1}[1+{r}_{x}(x,y)f(x,y)],$$

5 $${{{{{{\rm{M}}}}}}}_{y}(x,y)={c}_{2}[1+{r}_{y}(x,y)f(x,y)].$$

Pixel remapping operations as per the above maps are performed for both input tiles T1 and T2, and the resultant images are obtained as T1^*U*^ and T2^*U*^, respectively. For an estimated overlapping region of interest (ROI) R_12_, a parameter *d*_*k*_ is evaluated as follows, where *k* denotes each individual case while varying the distortion parameters, each within a specified range. Following the minimum-*d*_*k*_ case, the distortion parameters A, X_*off*_, and Y_*off*_ are obtained, and ${{{{{{\rm{M}}}}}}}_{x}(x,y)$ and ${{{{{{\rm{M}}}}}}}_{y}(x,y)$ are set fixed for subsequent pixel remapping operations.

6 $${d}_{k}=\frac{\mathop{\sum}\limits_{{{{{{\rm{All}}}}}}\,{{{{{{\rm{R}}}}}}}_{12}{{{\rm{pixels}}}}}\left|{{{{{\rm{T}}}}}}{1}^{U}-{{{{{\rm{T}}}}}}{2}^{U}\right|}{Area({{{{{{\rm{R}}}}}}}_{12})},$$

To assess the model, we simulated a grid image and induced an asymmetric radial distortion. Supplementary Figure 1a shows two of such identically distorted tiles. A straightforward mosaic-stitching is depicted in Supplementary Fig. 1b, where the red-marked ROI indicates a severe structural discontinuity. The distorted tiles in Supplementary Fig. 1a were processed with our proposed method, and the distortion parameters A, X_*off*_, and Y_*off*_ were estimated to be 51˚, −49, and −47, respectively, where a negative sign denotes an underlying flipping operation. It is noted that to apply the distortion effect to all 4 quadrants of the tiles, we propose flipping the inputs along the X axis, Y axis, and both X & Y axes, and repeating the pixel remapping operations based on the same maps as depicted in Eqs. (4) and (5). ${{{{{{\rm{S}}}}}}}_{x}$ and ${{{{{{\rm{S}}}}}}}_{y}$ were set at 60.0 throughout the analysis. The distortion-compensated tiles are depicted in Supplementary Fig. 1c. The curve lines become straight after the proposed correction. Supplementary Figure 1d shows a mosaic-stitched result with the distortion-compensated tiles, where the red-marked ROI reveals a near artifact-free nature.

The same approach was followed to one-time estimate the distortion parameters for our specific optical configuration. A typical H&E-stained tissue was imaged, collecting THG and TPEF signals from the H and E dyes, respectively. The proposed method was applied to two sets of vertically and horizontally acquired adjacent tiles. Each set consists of 4 continuous tiles, and thus provides 3 overlapping regions. It is noted that each tile was in 8-bit grayscale format. To make the process less susceptible to high-frequency noise, a morphological opening operation was performed on each input tile, followed by a binary thresholding operation. As shown in Supplementary Table 1, the average values of A, X_*off*_, and Y_*off*_ were found to be 25.2°, 6.0, and 20.0, respectively. Following the same, for all our experiments, we pre-calculate the pixel-coordinate maps ${{{{{{\rm{M}}}}}}}_{x}(x,y)$ and ${{{{{{\rm{M}}}}}}}_{y}(x,y)$ to assist with a near-instantaneous distortion compensation.

### Description of the-RSTS protocol

A Pasteur pipette was utilized to drop the relevant chemical solutions/liquids into a tissue chamber while allowing to nearly dip the tissue under the relevant liquid. While moving from one step to another, a tissue paper was utilized to quickly absorb the liquid inside the chamber. Supplementary Table 2 enlists the steps involved in the-RSTS protocol. In step 1, a 10% neutral buffered formalin solution (H121-08, Macro) was dropped and left for 20 s to yield a short fixation. Steps 2 and 3 involved two phases of H-staining enhancing the nuclei contrast. We first employed Gill’s No. 3 hematoxylin solution (GHS332, Sigma-Aldrich) for 1 min and 30 s, followed by Mayer’s hematoxylin solution (MHS16, Sigma-Aldrich) for 2 min and 30 s. In step 4, distilled water was used to rinse the tissue twice, taking 5 s each. Step 5 involved an ammonia solution (9721-1, J.T.Baker) for 15 s that served as a bluing reagent. Step 6 was to perform E-staining to express the vessels, fibers, and other relevant morphologies. Eosin Y (3800, J.T.Baker) was dropped on the tissue and left for 15 s. In the final step 7, 90% alcohol was used for 10 s to help wash out the excessive staining. A simple demonstration of all the steps 1–7 is provided in Supplementary Movie 1. It is noted that the tissue chamber utilized a 1-mm-thick *iSpacer* (SunJin Lab Co., Taiwan) attached to a typical microscope slide, providing a volumetric space of 16 × 26 × 1 mm^3^. Thus, the recommended quantity of each staining solution is around 0.4 ml. The recommended tissue-area ranges from 1 mm^2^ to 1 cm^2^, where the tissue-thickness is recommended to be in the range of 1 mm to 2 mm. Please note that owing to the toxic and cancerogenic nature of formalin, it is recommended to perform the-RSTS protocol with caution, preferably under a laboratory hood.

### Real-time color remapping to assist with a traditional histopathological visualization

Although a multi-channel mNLOG image with pseudo colors assigned to all the channels can help visualize or categorize channel-specific morphologies, the same might not be very convenient for a pathologist to conduct a rapid assessment. Therefore, our parallel attempt was to assist with a traditional histopathological visualization of our mNLOG images. A related prior study^51^ demonstrated a virtual H&E transillumination idea in the context of epi-fluorescence microscopy, where the Beer-Lambert law was employed as a fundamental basis. Applying the same theory in our mNLOG platform, we create two look-up tables (LUTs) for color remapping of THG and TPEF channels so as to resemble a typical H&E-specific image. To comprehend the idea, let us first define our desired blue, green, and red color compositions for the hematoxylin and eosin dyes to be (B_*H*_, G_*H*_, R_*H*_) and (B_*E*_, G_*E*_, R_*E*_), respectively. Presuming an 8-bit image, for an intensity range of $i:\,0-255$, we calculate the LUTs as

7 $${{{{{{\rm{LUT}}}}}}}_{H}\to \left\{\begin{array}{c}Blue\to {e}^{-(255-{{{{{{\rm{B}}}}}}}_{H})ik/255}\\ Green\to {e}^{-(255-{{{{{{\rm{G}}}}}}}_{H})ik/255}\\ Red\to {e}^{-(255-{{{{{{\rm{R}}}}}}}_{H})ik/255}\end{array}\right.\,{{{{{\rm{and}}}}}}\,{{{{{{\rm{LUT}}}}}}}_{E}\to \left\{\begin{array}{c}Blue\to {e}^{-(255-{{{{{{\rm{B}}}}}}}_{E})ik/255}\\ Green\to {e}^{-(255-{{{{{{\rm{G}}}}}}}_{E})ik/255}\\ Red\to {e}^{-(255-{{{{{{\rm{R}}}}}}}_{E})ik/255}\end{array}\right.,$$

where LUT_*H*_ and LUT_*E*_ are for hematoxylin and eosin channels, respectively, and the parameter *k* can be optimized to enable the desired contrast level. Note that the idea of creating these LUTs helps simplify per-pixel operations and assists us with a near-instantaneous CUDA-accelerated colormap conversion, which can be expressed as

8 $$m{{{{{{\rm{NLOG}}}}}}}_{THG}(r,c)\mathop{\longrightarrow }_{{{{{{{\rm{LUT}}}}}}}_{H}}^{{Color\,mapping}}{{{{{{\rm{H}}}}}}}_{remap}(r,c),$$

9 $$m{{{{{{\rm{NLOG}}}}}}}_{TPEF}(r,c)\mathop{\longrightarrow }_{{{{{{{\rm{LUT}}}}}}}_{E}}^{{Color\,mapping}}{{{{{{\rm{E}}}}}}}_{remap}(r,c),$$

where, $m{{{{{{\rm{NLOG}}}}}}}_{THG}(r,c)$ and $m{{{{{{\rm{NLOG}}}}}}}_{TPEF}(r,c)$ denote our THG and TPEF channel images, respectively, and ${{{{{{\rm{H}}}}}}}_{remap}(r,c)$ and ${{{{{{\rm{E}}}}}}}_{remap}(r,c)$ are the respective hematoxylin- and eosin-specific remapped images. Note that *r* and *c* respectively denote vertical and horizontal pixel coordinates. Subsequently, the remapped images in Eqs. (8) and (9) are combined as follows to obtain the final H&E remapped output as ${{{{{{\rm{HE}}}}}}}_{remap}(r,c)$.

10 $${{{{{{\rm{HE}}}}}}}_{remap}(r,c)={{{{{{\rm{H}}}}}}}_{remap}(r,c)\times {{{{{{\rm{E}}}}}}}_{remap}(r,c)/255.0$$

To observe the effectiveness of the proposed algorithm, we considered multiple H&E-stained tissue slides prepared from skin and brain specimens. We performed both the transmission light microscopy and the proposed mNLOG imaging, followed by a denoised-contrast-enhanced color-remapping. In each case, our color-remapped mNLOG output closely resembled the respective transmission light microscopy image. See Supplementary Fig. 2 for a detailed demonstration. The time complexity for color-remapping a 6000 × 6000-pixel tile was ~10 ms with CUDA acceleration. It is noted that the values of (B_*H*_, G_*H*_, R_*H*_) and (B_*E*_, G_*E*_, R_*E*_) were set as (180, 0, 90) and (210, 165, 250), and the value of *k* was set as 2.5. All parameters were decided based on visual response and suggestions from the pathologist.

### Digital boost **via** Denoised Contrast Enhancement

To comprehend the need for digital enhancement, we first demonstrate a simple example. Supplementary Figure 3a depicts an accumulation-free cropped mNLOG image of a human brain specimen. The tissue was applied with the-RSTS protocol prior to imaging. As usual, red and green channels in Supplementary Fig. 3b, c denote THG and TPEF signals originating from hematoxylin and eosin dyes, respectively. As our goal is to closely resemble a typical histology image, the above color-remapping algorithm was directly applied to the image in Supplementary Fig. 3a, and the result is depicted in Supplementary Fig. 3d. However, we could observe a strong background signal that resulted in poor structural visibility. This observation was consistent with our previous whole-mount staining study^35^. Nevertheless, for our fresh human brain tissues in this study, the issue was more prominent. It is noted that owing to an extra softness of unfixed brain tissue and further being focused on a short staining time, it was not feasible to thoroughly wash out the excess or residual staining. As a result, both THG and TPEF channels encountered a moderate to strong background, eventually degrading the visibility of the cellular morphologies after color-remapping.

To resolve this issue, we employed one of our recently published methods called Denoised Contrast Enhancement (DCE)^52^. Unlike alternative state-of-the-arts, such as contrast-limited adaptive histogram equalization, DCE first suppresses the background and then selectively optimizes the contrast of the structural details. Besides, DCE utilizes CUDA acceleration and takes only a few tens of milliseconds to process a 6000 × 6000-pixel image, allowing us to implement the same in real-time. It is noted that DCE provides a single-parametric control for user-friendly operation. The single adjustment parameter is denoted as ${\alpha }_{max}$. For application, performance, and additional details regarding DCE, see our previous publication^52^. In this present study, the typically used and recommended values of ${\alpha }_{max}$ for TPEF and THG channels are 5.0 and 8.0, respectively, which can be further optimized based on visual response. Demonstrating the effectiveness, Supplementary Fig. 3e–g depict the DCE-applied version of the original mNLOG image as shown in Supplementary Fig. 3a–c. A post-DCE color-remapping was performed, and the result is shown in Supplementary Fig. 3h, revealing a dramatically improved visibility of the cell nuclei and other tissue morphologies. A further improved version is depicted in Supplementary Fig. 3i–l with an optional post-DCE gamma correction followed by bilateral filtering prior to color-remapping. It is noted that the images depicted in Supplementary Fig. 3h, l closely resemble a typical histology image, where the purple-colored cell nuclei and pink-colored relevant morphologies are readily identifiable with an excellent contrast ratio. A simple comparison of the results in Supplementary Fig. 3d with Supplementary Fig. 3h, l clarifies the drastic improvement via DCE.

### A custom bitmap for ultra-large gigapixel datasets

While maintaining a large FOV-resolution ratio, a large number of pixels is always required to secure a high NFOM. For instance, if we target imaging a 1 cm^2^ area with an ultrafine pixel size of 167 nm (i.e., each 1 mm^2^ tile with 6000 × 6000 pixels), a total of 3.6 Gigapixels would be acquired in the process. Again, considering three channels, each pixel would take 24 bits, thus leading to 10 GB of data on disk without compression. It is noted that typical TIFF and PNG formats though they support compression, are not quite practical to store and display such huge datasets once processing speed becomes a primary concern. Therefore, we store the mosaic-stitched image as a custom bitmap that can support an ultra-large dataset with minimal time complexity, provided enough system memory is available. The bitmap file starts with a set of specific headers comprising acquisition information, such as image dimensions, number of tiles, etc., followed by a 10× downscaled version of the ultra-large image to enable a quick low-resolution preview. In the custom bitmap, data was organized in B, G, and R sequence, i.e., each pixel corresponds to three 8-bit values depicting the blue, green, and red channels, sequentially. It was noted that while employing a state-of-the-art NVMe SSD, a 1 Gigapixel 2.8 GB custom bitmap could be loaded and displayed in less than 2 s (see Supplementary Movie 2). It is noted that owing to the limited resolution of an off-the-shelf display screen, we implemented real-time crop and digital magnification features as demonstrated in Supplementary Movie 3 and Supplementary Movie 5.

### Statistics and reproducibility

In the clinical study, we considered the-RFP datasets for 50 normal and tumor-specific specimens (obtained from 8 participants) with their respective FFPE-biopsy images for the blind test. For both the-RFP and FFPE methods, the binary decisions (tumor versus normal) for each case were compared. Accuracy, sensitivity, and specificity are respectively obtained as follows:

11 $${{{{{\rm{accuracy}}}}}}=\frac{{{{{{\rm{TP}}}}}}+{{{{{\rm{TN}}}}}}}{{{{{{\rm{TP}}}}}}+{{{{{\rm{TN}}}}}}+{{{{{\rm{FP}}}}}}+{{{{{\rm{FN}}}}}}},$$

12 $${{{{{\rm{sensitivity}}}}}}=\frac{{{{{{\rm{TP}}}}}}}{{{{{{\rm{TP}}}}}}+{{{{{\rm{FN}}}}}}},$$

and

13 $${{{{{\rm{specificity}}}}}}=\frac{{{{{{\rm{TN}}}}}}}{{{{{{\rm{TN}}}}}}+{{{{{\rm{FP}}}}}}},$$

where TP, FP, TN, and FN denote true positive cases, false positive cases, true negative cases, and false negative cases, respectively.

### Reporting summary

Further information on research design is available in the Nature Portfolio Reporting Summary linked to this article.

## Results

### Post-processing-free mesoscale Nonlinear Optical Gigascope platform with rapid artifact-compensated 2D large-field mosaic-stitching (rac2D-LMS) approach

**Fig. 1:**
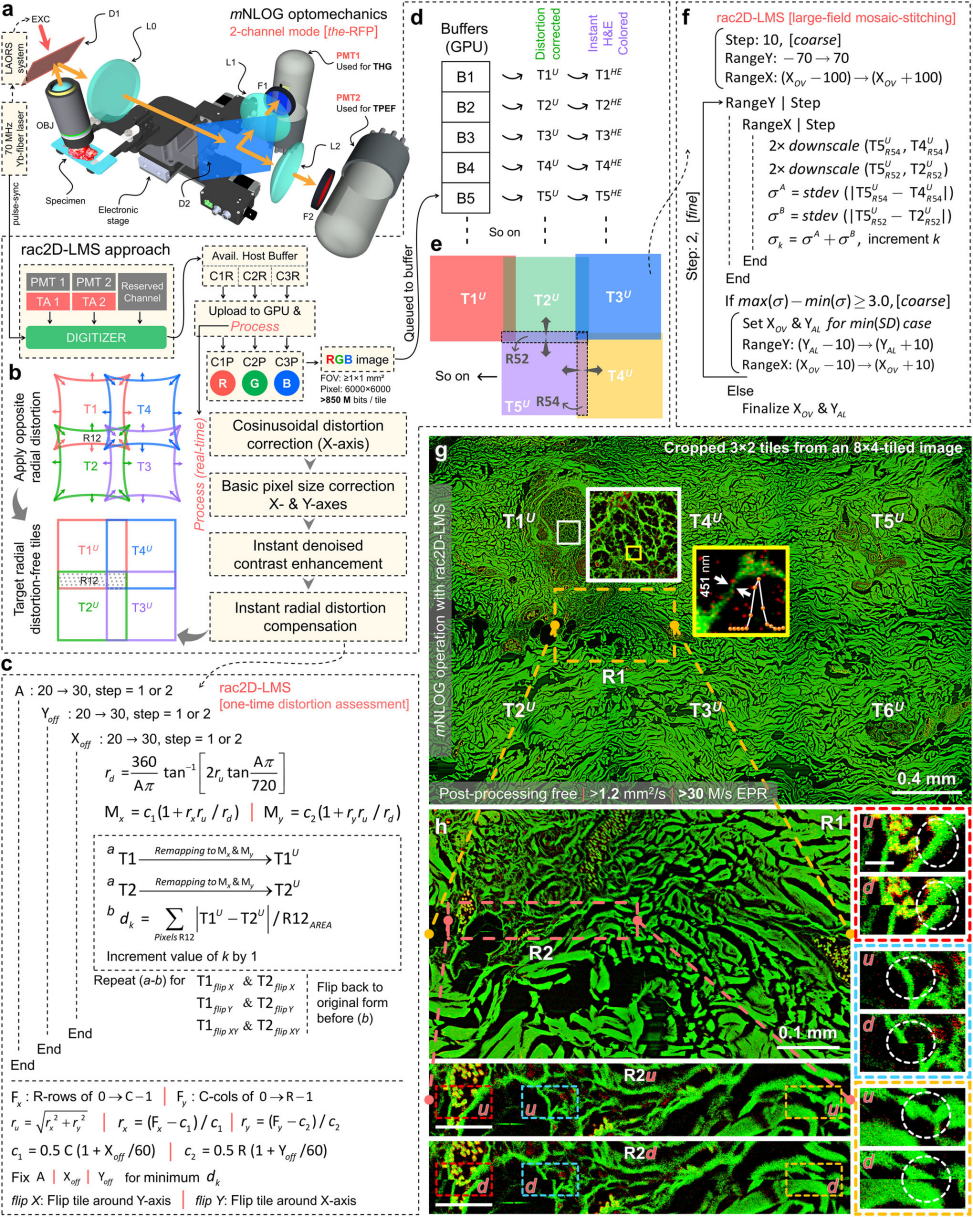
Post-processing-free mesoscale Nonlinear Optical Gigascope (mNLOG) platform assisted by rapid artifact-compensated 2D large-field mosaic-stitching (rac2D-LMS) approach. **a** 2-channel optical detection unit; LAORS: large-angle optical raster scanning, OBJ objective lens, EXC excitation beam, D1-2 dichroic beam splitters, L0-2 focusing lenses, PMT1-2 photomultiplier tubes, TA1-2 transimpedance amplifiers. Digitizer helps digitize TA1-2 outputs. Data undergoes real-time *Process* steps involving cosinusoidal distortion correction, pixel size calibration, denoised-contrast enhancement, and radial distortion compensation. The RGB image is generated. **b** The idea of distortion correction; for artifact-free structural continuity in R12, both T1 & T2 are expected to be distortion-free (T1^*U*^ & T2^*U*^). **c** One-time distortion assessment in rac2D-LMS; A, X_*off*_ & Y_*off*_ are control parameters, each varying within a specified range at a specified step. T1 & T2 are adjacent tiles with known overlap. T1^*U*^ & T2^*U*^ are the respective compensating distortion-induced tiles. Control parameters are chosen for the minimum *d*_*k*_ case, and coordinate maps M_*x*_ & M_*y*_ are set fixed. **d**, **e** Streamlined large-field multi-tile processing in rac2D-LMS. RGB images/tiles are queued to a set of pre-allocated buffers B1, B2, …, B200. T1^*U*^, T2^*U*^… & T1^*HE*^, T2^*HE*^… stand for distortion compensated and corresponding color-remapped versions, respectively. Queued tiles are sequentially processed via Compute Unified Device Architecture (CUDA)-acceleration to perform direct pixel/position-based spatial cross-correlation with a low-resolution coarse mode alignment followed by a high-resolution fine-tuning. **f** X_*OV*_ & Y_*AL*_ denote an estimated overlap & an off-axis alignment parameter, respectively. RangeY & RangeX are spatial ranges along vertical and horizontal axes, respectively. T5^*U*^ with major overlaps R54 and R52, as indicated in **e**, is taken as an example. Coarse mode involves a 10-pixel stepping, followed by a 2-pixel fine-tuning. **g**, **h** Demonstration of post-processing-free mNLOG imaging revealing an ultrahigh digital resolution and an artifact-compensated mosaic-stitching nature. **g** A 3 × 2-tile region cropped from a 1 Gigapixel mNLOG image (see the yellow-dashed box in Supplementary Fig. 4). T1^*U*^-T6^*U*^ are distortion-compensated tiles, scale bar: 0.4 mm. White boxed 570 × 570-pixel enlarged ROI depicts fine structural details, scale bar: 30 µm. Yellow boxed 63 × 59-pixel enlarged region of interest (ROI) resolves a 451 nm-thick ultrafine structure, scale bar: 3 µm. No optical zooming was employed. **h** Cropped and enlarged view of a 4-tile joining location R1 in **g**, scale bar: 0.1 mm. R2*u* & R2*d* show enlarged views of R2 in **h** with & without distortion compensation, respectively, scale bar: 40 µm. Red, cyan, and yellow ROIs in R2*u* & R2*d* are cropped and magnified alongside, scale bar: 10 µm. White-circled regions reveal an artifact-free mosaic-stitching nature; *u & d* stand for *undistorted* and *distorted* cases, respectively. Supplementary Movie 3 and 4 demonstrate an mNLOG imaging example and working of rac2D-LMS approach, respectively.

Effective scanning rate (ESR, mm^2^/s) can be defined as the true imaging speed considering all time penalties, such as but not limited to data acquisition, mechanical stage motion, mosaicking/stitching, color-remapping or additional relevant image/data processing, and displaying in full digital resolution. An optimum ESR is crucial for digitally assessing a centimeter scale area within a few tens of seconds. All associated image processing and mosaicking/stitching operations are thus expected to be real-time while maintaining a near artifact-free stitching quality and, most importantly, preserving the Nyquist-enforced pixel number. A 2-channel optical detection unit is depicted in Fig. 1a. An objective lens (OBJ) focuses an excitation beam (EXC, 70 MHz, 1070 nm) steered with a large-angle optical raster scanning (LAORS) system^37,49^ over a specimen placed on a motorized 3D stage. A dichroic beam splitter D1 reflects the emerging signal to a multi-channel detection unit comprising multiple relay systems with focusing lenses L0-2. Dichroic D2 is used for spectral separation, followed by band-pass filters F1-2 that help confirm the detection of specific signals of interest. Amplified PMT outputs from transimpedance amplifiers (TA1-2) are digitized. C1R-C3R in Fig. 1a denote available host buffers for 3-channel digitized data. C1R-C3R are uploaded to a CUDA-enabled graphics processing unit (GPU), and a *Process* is executed. In this study, C3R is reserved, and the demonstrations would be limited to 2-channel operation.

As illustrated in Fig. 1b, the *Process* first involves a cosinusoidal distortion correction owing to the nonlinear speed profile of a resonant scanner, followed by a pixel size calibration and a DCE (see “Methods” section). Calibrated image is processed with our proposed radial distortion correction idea. The principle is to introduce an appropriate opposite radial distortion to each tile so that the overlapping regions in two adjacent tiles become identical enough to assist with artifact-free mosaic-stitching. For a specific system configuration, to one-time assess the compensating radial distortion, we propose an approach as depicted in Fig. 1c, where two adjacent tiles (T1-2) are provided as inputs. Three distortion parameters are proposed, i.e., an angle (A, in degrees) governing the extent of distortion, and two spatial offsets (X_*off*_ and Y_*off*_**)** affecting the symmetry. For each case of A, X_*off*_, and Y_*off*_, each parameter within a specified range, the opposite distortion-induced outputs (T1^*U*^ and T2^*U*^) are obtained. Based on the sum of absolute differences for all pixels within the overlapping ROI (R12 in Fig. 1b, c), a parameter *d*_*k*_ is evaluated for each case. Values of A, X_*off*_, and Y_*off*_ are chosen corresponding to the minimum-*d*_*k*_ case. Following the same, pixel-coordinate maps ${{{{{{\rm{M}}}}}}}_{x}(x,y)$ and ${{{{{{\rm{M}}}}}}}_{y}(x,y)$ are calculated and set fixed for subsequent instant pixel remapping operations. See Supplementary Fig. 1 and Methods (Instantaneous distortion compensation model in rac2D-LMS) for detailed description and demonstration.

Processed versions of C1R-C3R are obtained as C1P-C3P, respectively. Designating a pseudo color to each 8-bit channel, we merge them into a 24-bit RGB image. Red and green colors would be used for THG and TPEF signals, respectively. Once the RGB image is ready, the same gets queued to the pre-allocated GPU buffers as shown in Fig. 1d. While an acquisition continues, the buffers B1, B2, …, B200 get occupied by distortion corrected RGB tiles T1^*U*^, T2^*U*^, and so on. Each queued tile enters a streamlined rendering in parallel to the high-NFOM mNLOG acquisition process. A simplified rendering example is shown in Fig. 1e, f.

T5^*U*^ is a tile to be aligned to the existing T1^*U*^-T4^*U*^ tiles. The major overlapping ROIs R54 and R52 are identified. Based on T4^*U*^ coordinates and displacement of the electronic stage, the extent of overlap or the R54-width (X_*OV*_) is roughly estimated. RangeY and RangeX are the spatial ranges along the Y and X axes, respectively. Aiming for a real-time operation, a coarse mode alignment is first rendered at 10-pixel stepping. For each T5^*U*^-step along X and Y, both R54 and R52 are evaluated. The R54-ROIs extracted from T5^*U*^ and T4^*U*^ are denoted as ${{{{{{\rm{T5}}}}}}}_{R54}^{U}(r,c)$ and ${{{{{{\rm{T4}}}}}}}_{R54}^{U}(r,c)$, respectively, each with R×C pixels. A 2× downscaling is applied, and a per-pixel absolute difference ${d}^{A}({r}^{//},{c}^{//})$ is calculated for all R54 pixels. Likewise, ROIs ${{{{{{\rm{T5}}}}}}}_{R52}^{U}$ and ${{{{{{\rm{T2}}}}}}}_{R52}^{U}$ are extracted for R52 and ${d}^{B}$ is obtained. Standard deviations ${\sigma }^{A}$ and ${\sigma }^{B}$ are calculated for ${d}^{A}$ and ${d}^{B}$, respectively, and a sum ${\sigma }_{k}$ is calculated. The maximum and minimum $\sigma$ values are obtained, and if the difference reaches a pre-defined threshold (typically ≥1.5), we render a fine-tuning following a reduced finer step size of 2 pixels. Note that Y_*AL*_ is defined to address an off-axis deviation of T5^*U*^ and T4^*U*^. X_*OV*_ and Y_*AL*_ would be simply replaced with Y_*OV*_ and X_*AL*_ for two vertically oriented tiles.

Streamlined real-time integration of rac2D-LMS with our high-NFOM multi-channel acquisition enabled a minimized host-GPU data overhead. rac2D-LMS utilized multiple asynchronous CUDA streaming, and considering our 6000 × 6000-pixel (864 M bit) tiles, average time complexities were found to be 3 ms for distortion correction, and <400 ms for a 2-tile mosaic-stitching operation. To demonstrate and validate the effectiveness, we performed a centimeter-scale gigapixel imaging of a standard H&E-stained tissue. Figure 1g depicts a mosaic-stitched view of 3 × 2 tiles (cropped from an ultra-large 8 × 4-tile image, see Supplementary Fig. 4). T1^*U*^-T6^*U*^ denote distortion-compensated tiles. Each tile is with an FOV of 1.1 × 1.1 mm^2^ comprising 6000 × 6000 pixels, ensuring a pixel size of 183 nm. It is to be noted that the original 8 × 4-tile image (see Supplementary Fig. 4) depicts an 8.1 × 4.1 mm^2^ imaging area that consists of a total of 1 Gigapixel and was acquired in 31 s, revealing a sustained effective throughput of >770 M bits/sec, including all time-penalties across the specimen-to-digital-display pixel pathway. To reveal our ultra-high digital resolution, the white marked ROI is enlarged, comprising submicron structures. For better visibility, another yellow marked ROI is selected, and the magnified view is depicted alongside. A 451-nm-thick ultrafine structure is digitally retrieved. To reveal our artifact-free mosaic-stitching quality (see Supplementary Movie 4), we mark a 4-tile joining location R1, and an enlarged view is shown in Fig. 1h, scale bar: 0.1 mm. For better visibility, R2 is marked and again magnified. R2 depicts an artifact-free structural continuity. R2*u* and R2*d*, respectively, show the results with and without distortion compensation, scale bar: 40 µm. Red, cyan, and yellow ROIs in R2*u* and R2*d* are cropped and magnified alongside, scale bar: 10 µm. White-circled regions in each case validate the effectiveness of our proposed method, where *u (undistorted)* and *d (distorted)* respectively denote with and without distortion compensation scenarios.

### The sub-6-minute True-H&E Rapid whole-mount-Soft-Tissue Staining (the-RSTS) protocol

Staining a specimen with traditional H&E dyes not only improves structural visibility but also makes our approach compatible with the global gold standard, and thus eliminates the necessity of additional interpretation training^29^. However, owing to the extra softness of unfixed brain tissue, it becomes highly frangible in nature, and one might therefore damage or disintegrate the tissue morphologies during a conventional whole-mount staining procedure^35^. Addressing this critical concern, the-RSTS protocol was introduced utilizing the traditional H and E dyes, yet consuming less than 6 minutes of cumulative processing time.

**Fig. 2:**
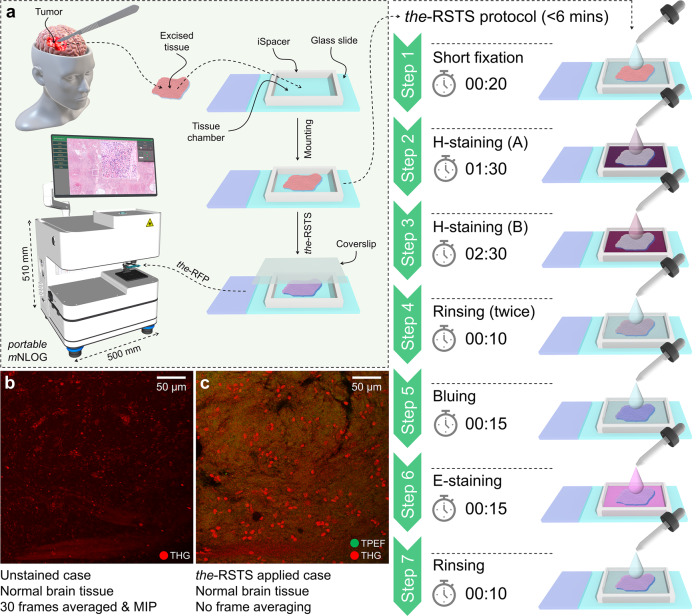
True-H&E Rapid Fresh digital-Pathology (the-RFP) procedure assisted by a sub-6-minute True-H&E Rapid whole-mount-Soft-Tissue Staining (the-RSTS) protocol. **a** Excised tissue is mounted in a chamber made of an *iSpacer* attached to a microscope slide. The-RSTS consumes <6 min of duration, which involves a short fixation, two phases of H-based nuclei staining, intermediate rinsing, bluing, E-staining, and final rinsing. Once the-RSTS is completed, a coverslip is added, and imaging is performed. Supplementary Movie 1 demonstrates the-RSTS procedure. **b**, **c** Effectiveness of the-RSTS protocol: **b** Z-projected image of an unstained normal brain tissue, where each slice was acquired via averaging of 30 frames, and **c** a single slice with no frame accumulation, acquired after performing the-RSTS procedure on the same specimen, revealing a drastic improvement to the cellular visibility. Scale bar: 50 µm. The red color in **b** and **c** and the green color in **c** depict third harmonic generation (THG) and two-photon excitation fluorescence (TPEF) signals, respectively. For more details on the sources of the-RSTS image contrast, see our spectroscopy analysis in Supplementary Fig. 5. For analysis of the autofluorescence signal, see Supplementary Fig. 6.

As illustrated in Fig. 2a, an *iSpacer* with a thickness of 1 mm was placed on top of a typical microscope slide yielding a small chamber. The excised brain specimen was placed inside this chamber, and the-RSTS protocol was performed. As shown alongside, steps 1 to 7 were executed sequentially. For a detailed description, see “Methods” section (Description of the-RSTS protocol). Once the-RSTS procedure was over, a coverslip was added that helped flatten the top surface of the specimen. Subsequently, the-RFP was performed with our developed mNLOG imaging platform, as shown in Fig. 2a. The dimension of the mNLOG platform is approximately 0.5 × 0.5 × 0.5 cubic meters, making it portable enough to be placed inside a surgical room. However, in the present design, the laser source is external, and therefore requires additional space. An inverted configuration of the set-up with an integrated laser source would be helpful for an even more compact system to enable user-friendly standalone operation.

It is to be noted that in the context of nuclei staining, Gill’s hematoxylin showed promising result, though the subsequent eosin staining quality was not that acceptable. On the other hand, in the case of Mayer’s hematoxylin, the subsequent eosin staining was way stronger; however, the visibility of the cell nuclei became poor. Therefore, both Gill’s & Mayer’s hematoxylin solutions were combined, followed by an E-staining to help improve the overall image quality. See Supplementary Fig. 7 for a detailed analysis of Gill’s and Mayer’s hematoxylins applied to fresh human brain specimens. To assess the effectiveness of the-RSTS protocol, we performed a simple experiment. A normal brain tissue was first imaged with no staining applied, and THG signal was collected. Figure 2b depicts a maximum intensity projected (MIP) image, where each slice was acquired via averaging of 30 frames. In this case, lipofuscin contents in the brain often contribute to the THG signal, differing from other THG-based reports on the high sensitivity on cellularity^46,47^. Nevertheless, such THG signals make cell detection or classification challenging, less reliable, and time-consuming, especially when we target a fast training-free solution. In the next step, the same tissue was applied with the-RSTS protocol, and imaging was performed again. As depicted in Fig. 2c, the-RSTS applied case shows a drastic improvement in cellular visibility, especially the cell nuclei. It is to be noted that the image in Fig. 2c is a single slice with no frame averaging, while each effective pixel signal was excited with only 1–2 laser pulse(s) with a pulse energy of <2 nJ. The imaging position before and after the-RSTS was approximately the same, though owing to the extra softness of the tissue, it was challenging to maintain a perfect match. Therefore, a Z-projection of 40 slices at an axial step of 1 µm is shown for the unstained case, whereas only a single imaging slice is depicted for the-RSTS applied scenario.

### Demonstration of True-H&E Rapid Fresh digital-Pathology technique with a centimeter-scale excised tumor specimen

**Fig. 3:**
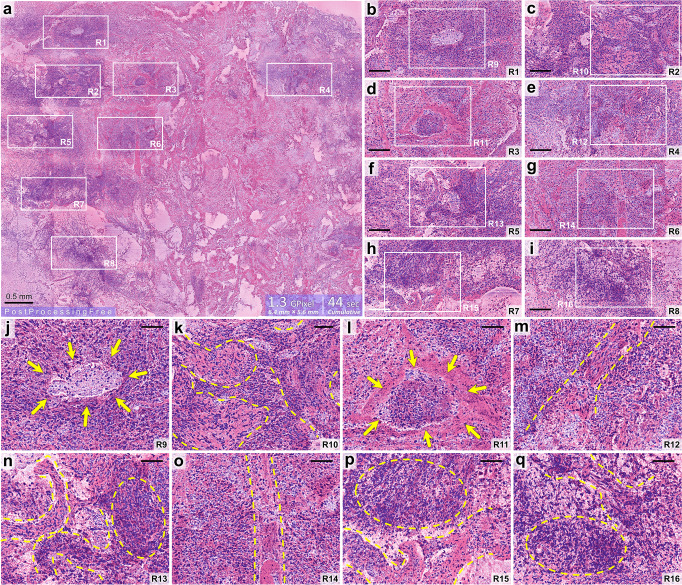
Demonstration of True-H&E Rapid Fresh digital-Pathology (the-RFP) approach via a centimeter-scale tumor tissue sample with zero post-acquisition data/image processing. **a** A 1.3 Gigapixel color-remapped the-RFP image, scale bar: 0.5 mm, field-of-view (FOV): 6.4 × 5.6 mm^2^, cumulative imaging time: 44 s, true effective-pixel-rate (true-EPR): ~30 M/s, true effective-scanning-rate (true-ESR): >1 mm^2^/s. **b**–**i** Cropped and enlarged regions of interest (ROIs) R1-8 marked in **a**, pixel number: 6700 × 3300, scale bar: 150 µm. **j**–**q** Cropped and enlarged ROIs R9-16 marked in **b**–**i**, pixel number: 3200 × 2500, scale bar: 70 µm. Distribution, shape, and size of the cell nuclei and other structural details, such as, the vessels, can be visualized in **b**–**q** as represented by the purple and pink morphologies. Yellow-dashed curves mark some vessel structures. Yellow-dashed circles show examples of hypercellular regions. Arrows in **j** mark a suspected necrotic region. Atypical cellularity is prominent inside some atypical vessels, such as, **k**, **m**, **n**, **q**, indicating signs of microvascular proliferation. Supplementary Movie 5 provides a post-processing-free subminute gigapixel demonstration of the-RFP technique.

To demonstrate the-RFP technique, we considered a centimeter-scale excised whole-mount tumor tissue sample. Utilizing H&E dyes, the-RSTS protocol was applied consuming 6 minutes of duration. Subsequently, multi-channel mesoscale high-NFOM acquisition was performed with our mNLOG imaging platform with enabled real-time DCE^52^ followed by real-time color-remapping. For additional information, see “Methods” section (Real-time color remapping to assist with a traditional histopathological visualization, and Digital boost via DCE). It is noted that DCE helps suppress strong backgrounds which might arise owing to leftover/excess staining, and the subsequent color-remapping operation helps our nonlinear optical images to be digitally displayed with the traditional histopathology colors and hues for accurate diagnosis. Figure 3a depicts an ultra-large image (the-RFP) comprising 1.3 Gigapixels, with an FOV of 6.4 × 5.6 mm^2^, scale bar: 0.5 mm. A pixel-size of ~167 nm was thus maintained, ensuring NFOM > 1. No frame averaging/integration was applied, and each tile/slice comprising 6000 × 6000 pixels was acquired in a single shot. It is to be noted that the 1.3 Gigapixel 6.4 × 5.6 mm^2^ image was acquired in just 44 s, which includes multi-channel data acquisition, calibration/distortion-correction, digital enhancement, color-remapping, mosaic-stitching, visualization/display with the full digital resolution, and most importantly includes the time-penalty owing to mechanical stage motion. A true effective-pixel-rate (true-EPR) of around 30 M/s, and a true-ESR of >1 mm^2^/s were preserved, revealing a dramatic improvement in comparison to the prior state-of-the-art label-free brain tumor assessment studies as enlisted in Table 2.

**Table 2:** A comparison of state-of-the-art label-free nonlinear optical imaging studies dedicated to human brain tumor assessment.

Refs.	FOV (µm^2^)	Pixel number	EPR (M/s)	ESR (mm^2^/s)	Digital resolution (µm)	Pixels in 1 cm^2^ area (×10^9^)	1 cm^2^ scan & display time (min)
^48^	350 × 350	1024 × 1024	0.11	0.013	0.7	0.9	>120
^47^	300 × 300	1000 × 1000	0.13	0.011	0.6	1.1	>120
^46^	450 × 450	1000 × 1000	0.12	0.023	0.9	0.5	>60
the-RFP (this report)	1000 × 1000	6000 × 6000	~30	>1	<0.5	3.6	2^a^ (scan) 6 (stain)

^a^Adjacent-tile overlap: 9% of the field-of-view (FOV); includes time penalties owing to mechanical stage motion, mosaic-stitching, color-remapping, and displaying in full resolution. This report involves a sub-6-minute True-H&E Rapid whole-mount-Soft-Tissue Staining (the-RSTS) protocol. Parameters cited/estimated are based on reported results and to the best of the author’s knowledge.

As Fig. 3a depicts a huge imaging area, adequate digital magnification is a must to visualize the microscopical details properly. Therefore, several ROIs R1-8 are marked inside Fig. 3a, each with 6700 × 3300 pixels. Cropped and enlarged views of R1-8 are shown in Fig. 3b–i, each with a scale bar of 150 µm. At this stage, we can observe the cellular details. For a more detailed visualization, another set of ROIs R9-16 are marked inside R1-8, sequentially. Cropped and enlarged views of R9-16 are depicted in Fig. 3j–q, each with a scale-bar of 70 µm. It is noted that the distribution, shape, and size of the cell nuclei can be clearly visualized from the magnified ROIs. Analogous to a typical histopathology image, the cell nuclei are represented in purple hue, while the other relevant morphological information, such as, the vessel structures, are depicted in pink. Substantial hypercellularity can be seen in multiple regions. For example, see the yellow-dashed circles in Fig. 3n, p, q. Arrows in Fig. 3j show an indication of necrosis. Atypical vessels are prominent, as marked by the yellow-dashed curves. In Fig. 3k, m, n, q, cellularity seems abnormal inside the vessels, indicating signs of microvascular proliferation. See Supplementary Movie 5 for a comprehensive demonstration of the-RFP technique.

**Fig. 4:**
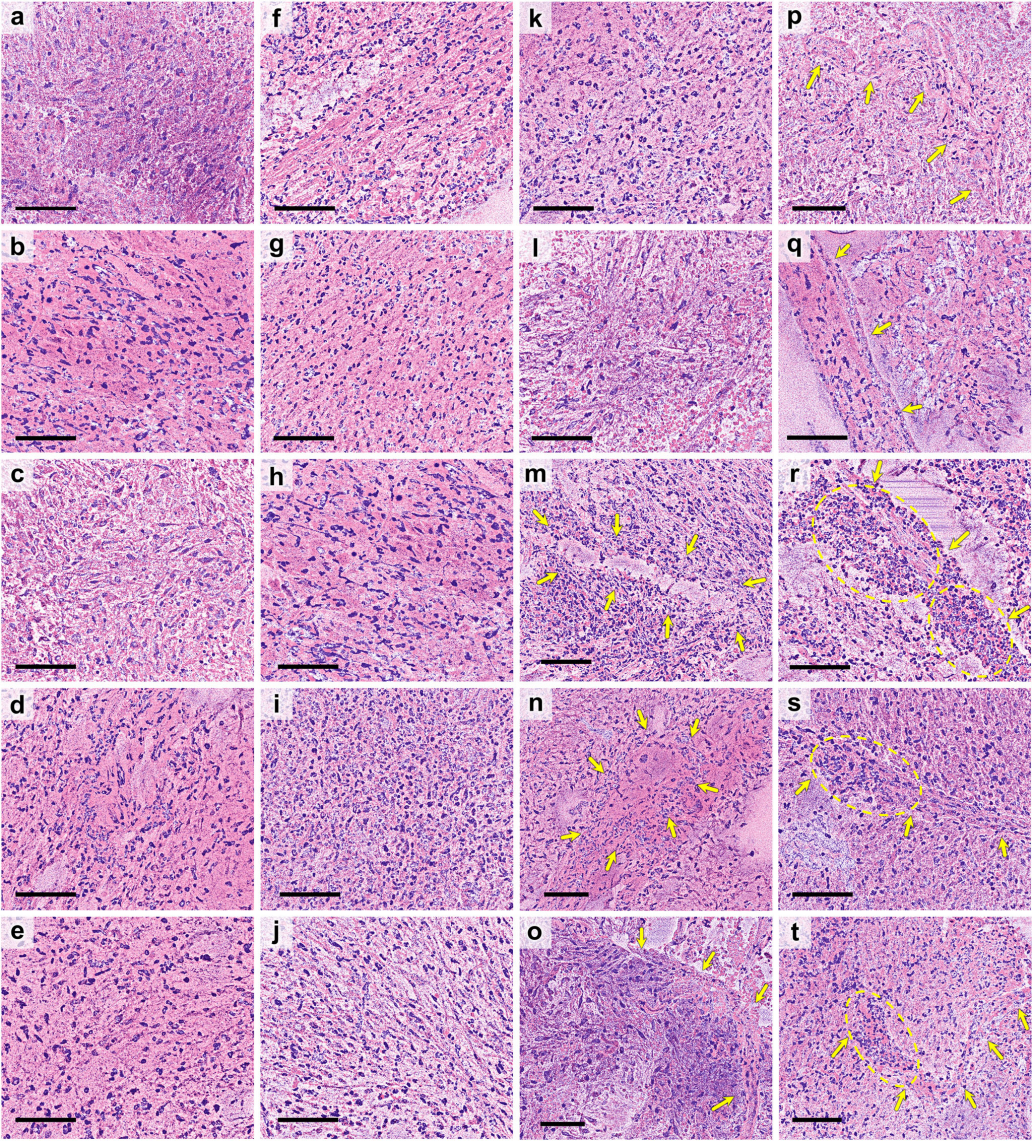
True-H&E Rapid Fresh digital-Pathology (the-RFP) images depicting typical tumor-specific features, scale bar: 100 µm. **a**–**t** Nuclear atypia and/or hypercellularity. The distribution, shape, and size of the nuclei seem inconsistent. **m**–**n** Suspected necrotic areas, where arrow-marked regions show much lower cellularity compared to the adjacent hypercellular regions. **o**–**t** Atypical vessels (arrow marked); see a typical vessel in Fig. 5a. Circled regions in (**r**–**t**) clearly show hypercellularity inside the vessels, suspecting microvascular proliferations. Additionally, an example of mitosis is depicted in Supplementary Fig. 8.

**Fig. 5:**
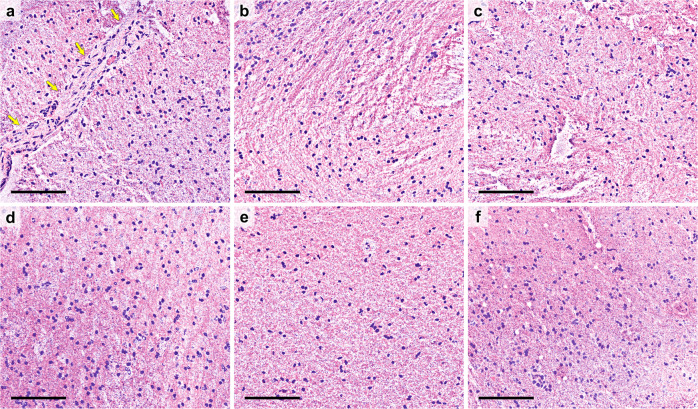
True-H&E Rapid Fresh digital-Pathology (the-RFP) images from normal brain specimens, scale bar: 100 µm. **a**–**f** Distribution of normal cell nuclei, where the overall cellularity, especially the nuclear shape and size seem consistent (see high cellularity and nuclear atypia in Fig. 4). Arrows in **a** mark a normal vessel with typical endothelial cells (see atypical vessels in Fig. 4r–t with suspected microvascular proliferation).

Considering the-RFP datasets from multiple specimens, Fig. 4 depicts several cropped ROIs indicating typical tumor features, each with a scale bar of 100 µm. Nuclear atypia and/or high cellularity are quite common in most of the cases in Fig. 4a–t. For example, in each case of Fig. 4a–e, we observe that the shapes and sizes of the nuclei vary atypically. Some of the nuclei are much bigger in size in comparison to the adjacent ones. Some nuclei are round-shaped, while some others are elliptical or with a highly elongated shape. For a comprehensive understanding, we present Fig. 5, where a few cropped the-RFP images are depicted from normal brain specimens, scale bar: 100 µm. Unlike the nuclei in Fig. 4a–e, in each case of Fig. 5a–f, the nuclei shapes and sizes are quite consistent. In addition, the density of nuclei (cellularity) is another critical factor while identifying a tumor. As evident, the nuclei density in case of the tumor-specific cases in Fig. 4 seems quite high in comparison to the normal cases in Fig. 5. Aside from high cellularity and nuclear atypia, the existence of abnormal vessels, microvascular proliferation, necrotic areas are important indications of an existing brain tumor. Suspected necrotic regions are seen in Fig. 4m, n. In each case, the arrows mark an area where the cellularity seems much lower compared to the hypercellular vicinity. In Fig. 4o–t, several atypical vessel structures can be visualized. Atypical cellularity can be clearly observed inside a few vessels, for instance, see Fig. 4r–t, which might be an indication of microvascular proliferation. To comprehend this point, we might compare it with a normal vessel, as marked in Fig. 5a, where the shapes and distribution of the typical endothelial cells should be noted. For a tumor-specific case of microvascular proliferation, atypical tumor cells often appear inside the vessel, as marked by the yellow-dashed circles in Fig. 4r–t.

### Accuracy and reliability assessment

To investigate the accuracy and reliability of the-RFP technique, a non-interventional non-inferiority blind clinical study (diagnostic) was conducted through the collaboration between the Division of Neurosurgery, Department of Surgery, and Department of Pathology of National Taiwan University Hospital. Tumor-specific specimens were collected from 4 glioblastoma (diffuse gliomas of WHO grade 4) patients undergoing tumor resection surgeries. To maintain a balance between the malignant and benign cases, normal brain specimens were further collected from preserved brain tissues from 4 participants. Thus, from a total of 8 participants, we considered 50 specimens in total. For additional information, see “Methods” section (The clinical study).

**Fig. 6:**
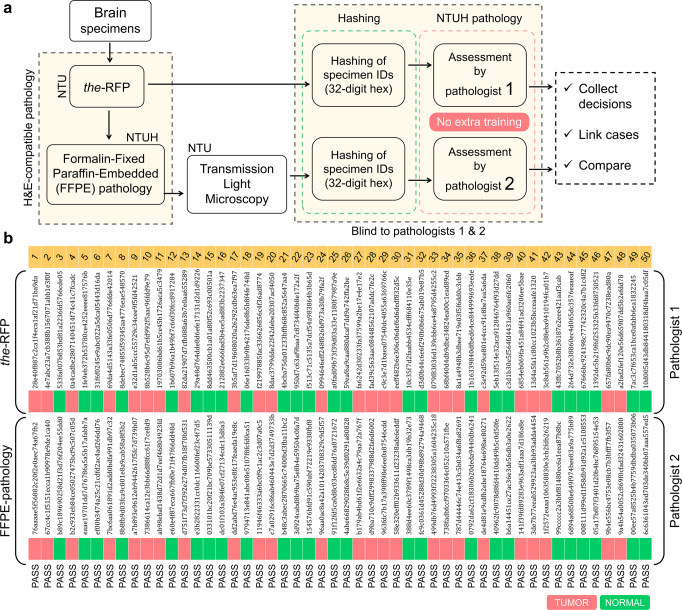
The process and outcome of the blind test (non-interventional). **a** Block diagram representation depicting each step involved. **b** Result of the blind test that includes a total of 50 specimens investigated through both True-H&E Rapid Fresh digital-Pathology (the-RFP) and formalin-fixed paraffin-embedded (FFPE)-biopsy procedures. Hashed IDs and respective outcomes are depicted for each case. The test concludes a 100% match between the-RFP and FFPE-biopsy decisions. Pathologists 1 and 2 did not receive any additional interpretation training prior to the assessments. See Supplementary Data 1 for the-RFP decisions and respective comments from the pathologist.

The block diagram representation in Fig. 6a depicts the entire protocol/process of the study. The collected brain specimens first underwent the-RFP procedure. Prior to mNLOG imaging, the-RSTS protocol was applied to each case. Once the image acquisition was over, the specimen was fixed in 10% neutral buffered formalin for at least 12 h. Each fixed specimen was transferred to the Department of Pathology, NTUH, Taiwan, and an FFPE biopsy was conducted. Multiple FFPE-biopsy slides were prepared for each specimen, and sent back to NTU. By means of a transmission light microscope, images were collected for the FFPE-biopsy slides. All 50 specimens underwent the same cycle. It is noted that since both the-RFP and FFPE-biopsy use the same gold standard H&E-dyes, the same chemical interaction is obvious. Therefore, the-RFP does not affect the quality of a subsequent FFPE biopsy (see Supplementary Fig. 9), and thus, no tissue washing was required in the process.

Subsequently, the-RFP and FFPE-histology datasets were assigned with two sets of independently generated random IDs, and sent to pathologist 1 (H.-Y. H.) and pathologist 2 (K. C.), respectively, to conduct assessments. No additional interpretation training was provided to any of the pathologists. The outcomes were collected (see Fig. 6b), and we observed that the-RFP decisions for all 50 specimens matched the FFPE pathology results, thus concluding an excellent accuracy comparable to the gold standard, revealing sensitivity, and specificity of both 100%.

## Discussion

The extent-of-resection (EOR) in a tumor surgery is the single most controllable factor that affects patients’ prognosis. EOR is often limited by concerns of injuring eloquent regions, especially in the case of a vital organ, such as the human brain or the central nervous system. Therefore, an ITA becomes a useful strategy so that the entire tumor can be effectively resected while not much sacrificing the nearby healthy regions. Depending on the situation, multiple rounds of ITAs might be necessary while making certain surgical decisions. Therefore, it is important that an ITA is performed as fast as possible so that the cumulative surgery time remains practical and safe for the patient.

In this paper, we introduce the-RFP, a WSSI digital ITA solution enabling 4× faster assessment compared to the global standard of FS-biopsy. The-RFP is compatible with the standard H and E dyes analogous to a traditional FS/FFPE-biopsy. To assist with the-RFP, a post-processing-free mNLOG platform was introduced that enables optical scanning and digital displaying of a 1 cm^2^ area with 3.6 Gigapixels in <120 s with a sustained >700 M bits/sec effective throughput. We made this possible, thanks to the introduction of our CUDA-accelerated high-performance rac2D-LMS approach streamlined to our large-FOV high-NFOM resonant raster-scanning and acquisition system. In this paper, our demonstration of the-RFP was dedicated to excised fresh human brain specimens to investigate tumor tissues. Specifically for soft and frangible fresh brain specimens, a sub-6-min protocol the-RSTS was implicated making use of traditional H&E dyes. Training-free blind assessments revealed 100% success in the identification of a tumor, indicating an FS/FFPE-comparable accuracy with sensitivity and specificity of both 100%. See Table 1 for a comparison with prior reported state-of-the-art ITA-capable technologies. Aside from excellent reliability, the-RFP apparatus comes in a small form factor that yields the potential to be placed inside an operating room. It thus might eliminate repeated transferring of specimens, thereby simplifying an ITA.

It is noted that a few of the prior reported physical sectioning-free fast-ITA approaches are capable of being applied in some specific surgical pathology applications^53,54^. Stimulated Raman microscopy^55^ provides useful chemical information to examine cellular anomalies; however, it often encounters a low signal-to-noise ratio (SNR) and might not be suitable for rapidly investigating a centimeter-scale area owing to its poor imaging speed. Optical coherence tomography^56^, despite providing high-speed imaging, encounters poor spatial resolution, poor SNR, and poor contrast ratio. Ultraviolet photoacoustic microscopy^28,57,58^ is another emerging modality in this field; however, the prior arts are often limited by effective throughput, and usually require machine learning and/or additional interpretation training. It is noted that light sheet fluorescence microscopy^59^, confocal microscopy^60^, and ultraviolet surface excitation^61^ microscopy are noteworthy approaches which might not work with dyes without fluorescence, such as the gold standard hematoxylin^36^. This is where a nonlinear higher harmonic generation becomes helpful to yield hematoxylin-driven nuclei contrast so as to make the assessment as reliable as a traditional FS/FFPE biopsy. Our group previously reported a nonlinear optical microscope (NLOM)^35^ with an H&E-staining protocol that is quite promising for reasonably firm tissues, such as skin specimens; however, the approach was limited by <0.1 mm^2^ FOV, a slow imaging speed, and lack of real-time stitching ability to meet the requirements of an artifact-free rapid ITA, not to mention the adoption of a Cr:forsterite laser for three-photon excitation of eosin.

In the context of human brain tumor assessment, a recent article^48^ revealed a 3-channel NLOM, that imaged cellular morphologies via TPEF signals from nicotinamide adenine dinucleotide (NADH) and flavin adenine dinucleotide (FAD) components, and utilized second harmonic generation (SHG) to reveal vessel structures. Two prior studies^46,47^ utilized THG to visualize cellular details, and SHG for expressing vessels. It is noted that for an analysis to be reliable, the acquired images must hold a sufficient digital resolution and an adequate SNR. Such label-free prior arts often employed prolonged accumulation of the respective signal of interest to secure a high SNR. As evident from Table 2, the prior arts employed several microseconds of effective pixel dwell time with a poor EPR of <0.2 M/s. A small FOV further resulted in a poor ESR of <0.025 mm^2^/s. Considering a 1 cm^2^ tissue area, such label-free approaches might consume up-to an hour or more which might be even longer than FS-biopsy, not to mention the reliability and interpretation concerns.

In a typical clinical practice, an ITA is often followed by an FFPE biopsy confirmation. Keeping this in mind, each specimen was sent for an FFPE biopsy after the completion of the-RFP procedure. No tissue washing or tissue removal was required or performed prior to FFPE procedure owing to the fact that both the-RFP and FFPE biopsy make use of the same gold standard H&E dyes. It is noted that for a tissue processed with certain non-H&E staining dye(s), specific washing steps might be necessary while undergoing a subsequent FFPE-biopsy. This is especially inconvenient for cases like our current study, where the samples are highly frangible, and the tissue volume is small to allow any possible waste.

It is to be noted that H&E is widely adopted for various surgical pathologies among all different surgical pathology levels^62^. With the-RFP approach, no additional interpretation training was required for or provided to any of the pathologists during the course of the blind assessments. In other words, the sources, H and E, of the image contrast enable the-RFP with excellent accuracy and reliability.

A common issue with any optical sectioning method is that to image an extended area without axial stepping, the tissue must be flat enough. Otherwise, one might need to acquire multiple slices at different depths to extract the morphologies. To minimize this issue, we opted for 1 mm thick *iSpacers* in the-RSTS procedure despite the fact that the specimens we considered were often thicker than 1 mm. As a result, when the stained specimen was mounted, the coverslip offered pressure and helped flatten the tissue.

As reported in this study, the cumulative time for staining, scanning, and displaying a 1 cm^2^ area is ~8 min, where the rapid staining procedure takes <6 min, and the subsequent scanning and display process consumes around 2 min of duration. The proposed staining time and selection of chemicals/liquids are primarily based on our visual perceptions from mNLOG imaging experiments. The cumulative assessment time can be further reduced with an even optimized staining protocol. Note that the current generation of rac2D-LMS as used in our mNLOG platform, consumes an average of <400 ms for our Nyquist-satisfied imaging tiles. It thus holds an ability to mosaic-stitch a ~ 12 × 12 mm^2^ area with a total of 130 G bits in 60 s, which is quite promising to be applied in a high-resolution WSI application^8,11,14^, not to mention with optimization of the relevant parameters, the processing time can be further reduced. Nevertheless, with our current 4 kHz nonlinear optical laser-scanning platform, 6000 slow-axis lines take ~0.8 s, which is twice the rac2D-LMS complexity. Therefore, it is fairly feasible to upgrade to an 8 kHz resonant scanning system without any frame-rate drop; however, it would require an even higher repetition-rate pulsed laser to maintain an adequate NFOM. With an 8 kHz upgrade, the-RFP effective throughout can be doubled to 1.4 G bits/s. Besides, our current electronic linear stages allow around 5.6 mm/s of traveling speed. Opting for alternative high-speed stages, such as X-LRM050 (Zaber Technologies Inc., Canada), would further help enhance the effective throughout. It is noted that, being a nonlinear laser scanning technology, the manufacturing cost of the mNLOG platform is higher than a typical linear imaging device. In this study, instead of using a chromium-forsterite laser^35^, we employed a typical fiber-based pulsed laser source to reduce the cost and to make the system compact and simple. In the near future, several off-the-shelf electronic components can be replaced with custom-designed electronics to minimize the cost of manufacturing.

Our experiments showed that the-RFP is capable of visualizing typical tumor characteristics, such as nuclear atypia, mitosis, microvascular proliferation, and necrosis to assist a pathologist with intraoperative assessment. It is noted that the tumor-specific brain specimens used in this study were collected from 4 patients with diffuse gliomas of WHO grade 4. To balance tumor-vs-normal cases, an equal number of preserved normal brain specimens were utilized from 4 participants. In daily clinical practice, tumor periphery is considered to be not representative of the tumor per se, and therefore cellular areas (generally at or near the tumor center) are mostly preferred for intraoperative examination. That is the reason why mixed tumor-normal regions were not considered in our clinical study. In the near future, further trials will be performed with diffuse gliomas of lower grades, in which the morphological distinction between tumor and normal regions may not be robust. In this study, despite the-RFP was demonstrated via imaging of excised human brain specimens, our technique holds the potential to be applied to other types of specimens, such as but not limited to breast, prostate, skin, etc., to make the relevant ITAs fast, accurate, and reliable. According to the World Health Organization, in the year 2020, around 2.3 million cases of breast cancer, 1.4 million cases of prostate cancer, and 1.2 million cases of non-melanoma skin cancer were newly identified across the globe. Especially for breast cancer, around 685,000 deaths occurred globally in the same year. Towards the end of 2020, globally, 7.8 million women were estimated to be living with breast cancer diagnosed in the past 5 years. The-RFP technique thus holds the potential to be readily extended to the surgical pathology associated with such most prevailing cancers. With rapid and accurate assessments provided by the-RFP, the effectiveness of such surgical procedures can be substantially enhanced, which would indeed improve patients’ prognosis and survival.

## Supplementary information

Supplementary Data 1

Supplementary Information

Supplementary Movie 1

Supplementary Movie 2

Supplementary Movie 3

Supplementary Movie 4

Supplementary Movie 5

Description of Additional Supplementary Files

Reporting Summary

## Data Availability

The data/images generated and/or analyzed to support the findings of our study are presented in the paper and the supplementary information. More details would be available from the corresponding author upon reasonable request.

## Code availability

A free version of the compiled standalone control and data acquisition software^49^ (*LASERaster*^*+*^) in the form of object codes with enabled stable features would be available from the corresponding author upon reasonable request, for non-commercial use only, subject to regulations of National Taiwan University, Taiwan.
