# Supplementary material for: Rapid digital pathology of H&E-stained fresh human brain specimens as an alternative to frozen biopsy
Source: Commun Med (Lond). 2023 May 30;3:77. doi: 10.1038/s43856-023-00305-w (PMC10229595; doi:10.1038/s43856-023-00305-w)
Supplement: Supplementary file 2 — Supplementary Information [file 43856_2023_305_MOESM2_ESM.pdf]

Supplementary Information

Rapid digital pathology of H&E-stained fresh human brain specimens as an alternative to frozen biopsy

Bhaskar Jyoti Borah[1], Yao-Chen Tseng[1], Kuo-Chuan Wang[2,*], Huan-Chih Wang[2], Hsin-Yi Huang[3], Koping Chang[3], Jhih Rong Lin[3], Yi-Hua Liao[4], and Chi-Kuang Sun[1,5,6,*]

[1]*Department of Electrical Engineering and Graduate Institute of Photonics and Optoelectronics, National Taiwan University, Taipei, Taiwan.*

[2]*Division of Neurosurgery, Department of Surgery, National Taiwan University Hospital, Taipei, Taiwan.*

[3]*Department and Graduate Institute of Pathology, National Taiwan University Hospital, Taipei, Taiwan.*

[4]*Department of Dermatology, National Taiwan University Hospital and National Taiwan University College of Medicine, Taipei, Taiwan.*

[5]*Graduate Institute of Biomedical Electronics and Bioinformatics, National Taiwan University, Taipei, Taiwan.*

[6]*Molecular Imaging Center, National Taiwan University, Taipei, Taiwan.*

[*]*Corresponding authors: wang081466@ntuh.gov.tw; sun@ntu.edu.tw*

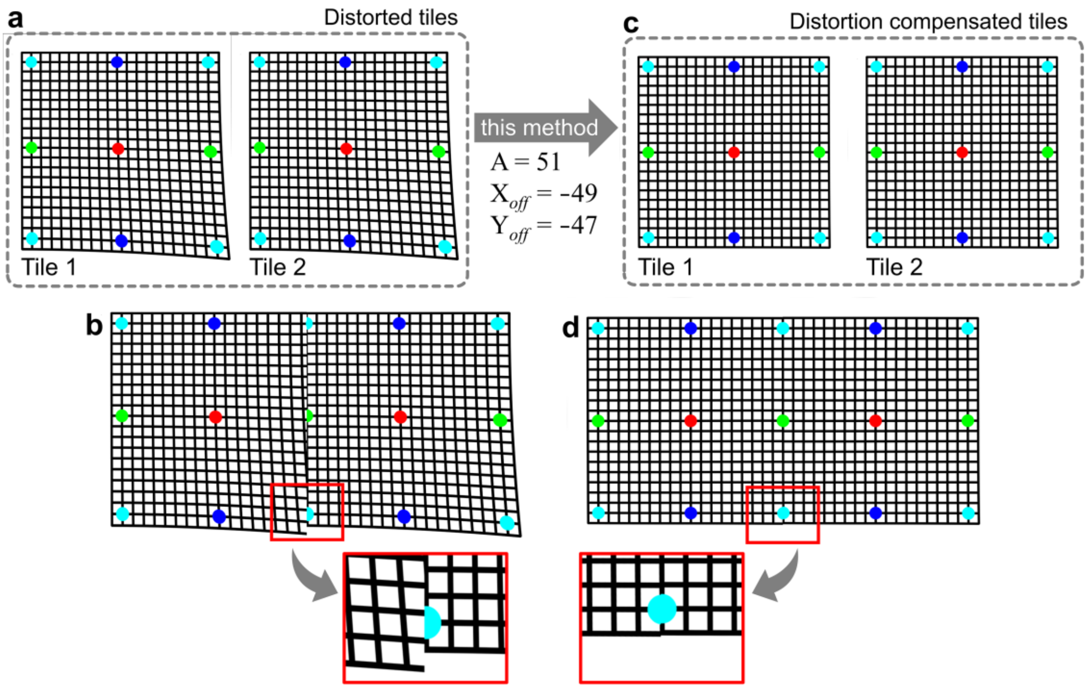

Supplementary Fig. 1 Demonstration of the proposed distortion compensation idea via simulated grid images. **a** Two asymmetrically distorted identical tiles, **b** straightforward mosaic-stitched view of the distorted tiles in (**a**). **c** Distortion compensated tiles obtained with our proposed method, where distortion parameters A, X_{off}, and Y_{off} were found to be 51˚, −49, and −47, respectively. **d** Mosaic-stitched view of the undistorted tiles in (**c**). Enlarged red-marked regions of interest (ROIs) in (**b**) & (**d**) reveal the effectiveness of our proposed distortion compensation method.

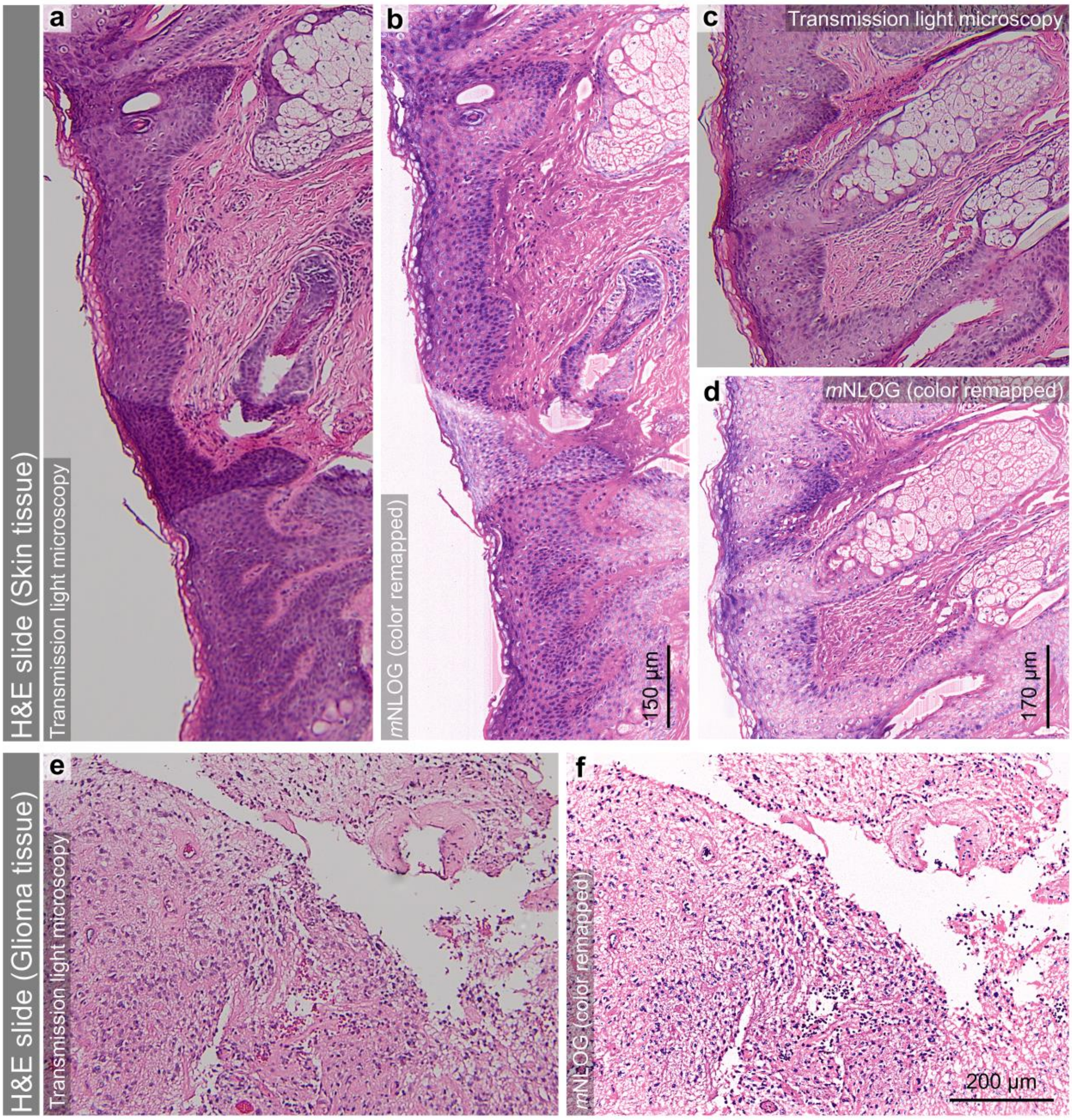

Supplementary Fig. 2 Demonstration of color-remapped images obtained from our mesoscale Nonlinear Optical Gigascope (mNLOG), resembling typical histopathology results. **a**, **c** Transmission light microscopy images of a standard H&E-stained slide (skin tissue). **b, d** Color remapped mNLOG images of the same slide at the respective positions shown in (**a**) & (**c**). **e** Transmission light microscopy image of a second H&E-stained slide (glioma tissue). **f** Color remapped mNLOG output for the same region in (**e**). In each case, our color-remapped mNLOG output closely resembled the respective transmission light microscopy image. Denoised contrast enhancement (DCE) was applied to each relevant mNLOG image prior to color remapping. Note that each mNLOG image is a single optical section, and no axial stacking or projection was employed. Refer to our previous publications[1,2] for lateral/axial point spread function (PSF) analysis. Scale bars: (**a** & **b**) 150 µm, (**c** & **d**) 170 µm, and (**e** & **f**) 200 µm.

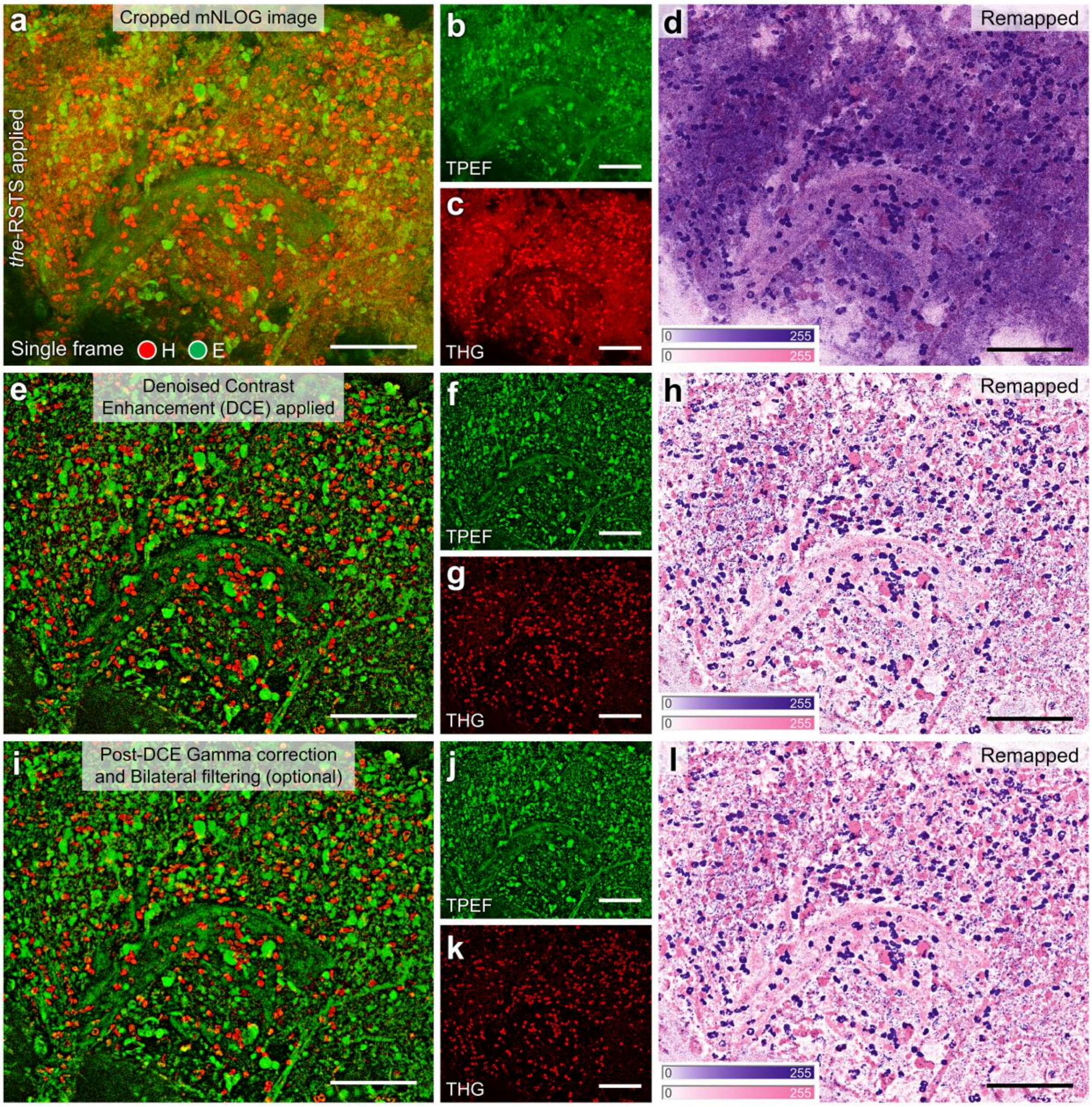

Supplementary Fig. 3 Demonstration of Denoised Contrast Enhancement (DCE)[3]-assisted color remapping. **a**-**c** An accumulation-free cropped image of a human brain specimen obtained from our mesoscale Nonlinear Optical Gigascope (mNLOG). True-H&E Rapid whole-mount-Soft-Tissue Staining (the-RSTS) protocol was applied prior to imaging. Red and green channels express third harmonic generation (THG) and two-photon excitation fluorescence (TPEF) signals originating from hematoxylin (H) and eosin (E) dyes, respectively. **d** A straightforward color-remapped version of (**a**) revealing a strong purple background obstructing structural visibility. **e**-**g** DCE-applied version of (**a**) with suppressed background in each channel. **h** Post-DCE color-remapped version of (**a**) revealing a dramatically improved visibility of the cell nuclei and other relevant morphologies. **i**-**l** A further improved version of (**d**) with an optional post-DCE gamma correction followed by bilateral filtering prior to color-remapping. Scale bar: 100 µm.

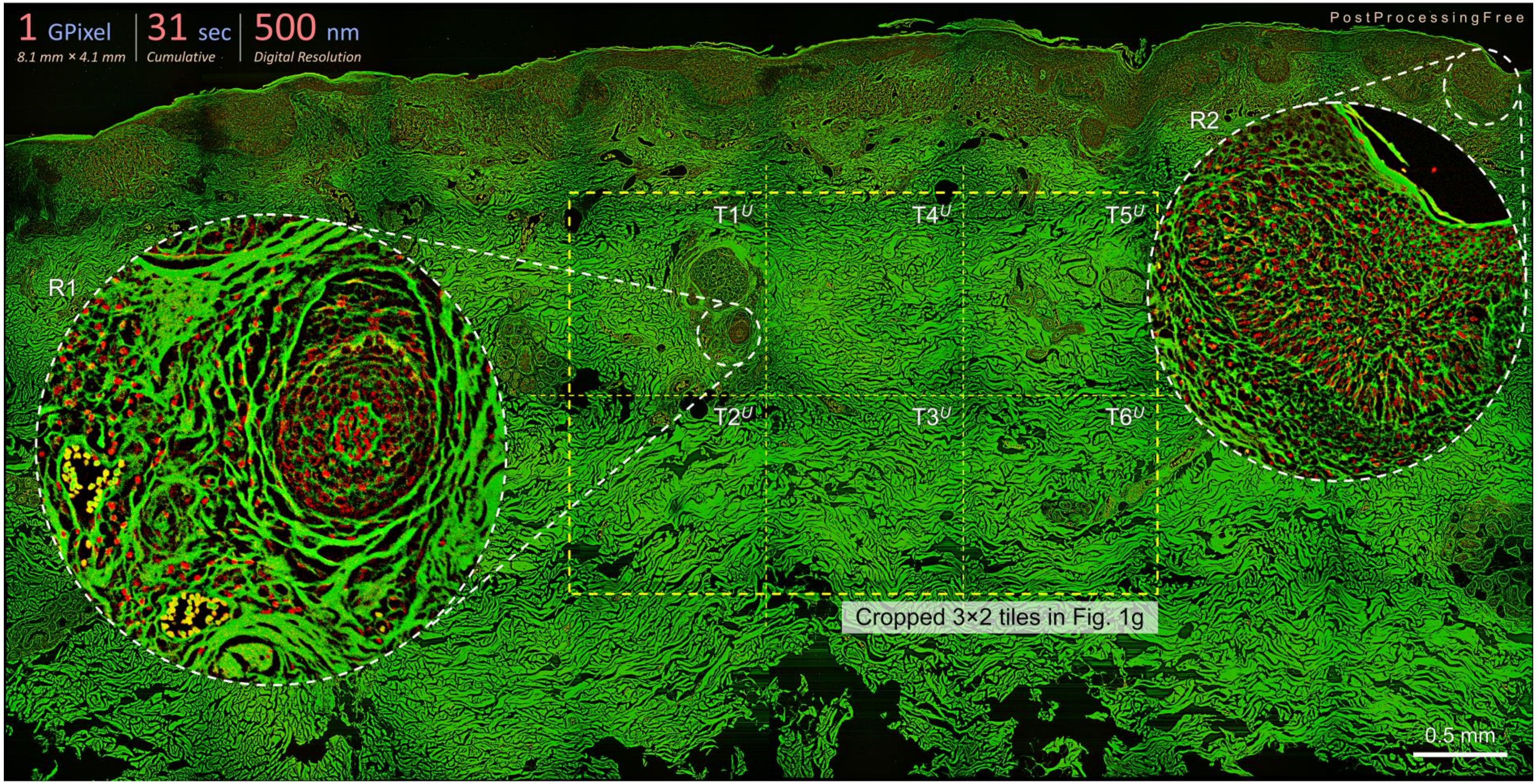

Supplementary Fig. 4 A 1 Gigapixel 8×4-tile laser-scanned nonlinear optical image depicting an 8.1×4.1 mm^2 (44,470×22,609 pixels) imaging area, which is acquired, mosaic-stitched, and displayed in cumulative 31 seconds. No post-acquisition data processing was involved. A true effective pixel rate (EPR) of >30 M/s and an effective scanning rate (ESR) of >1 mm^2/s were secured with a sustained effective throughput of >770 M bits/sec. A typical H&E-stained tissue slide (superficial basal cell carcinoma) was used as a sample. Each 1.1×1.1 mm^2 tile comprises 6000×6000 pixels (24-bit), ensuring a pixel size of 183 nm. Enlarged R1 (a hair follicle) and R2 (a tumor nest) reveal an optical-zoom-free excellent digital resolution. The yellow dashed box comprising 3×2 distortion compensated tiles (T1^{U}-T6^{U}) was cropped to Fig. 1g for demonstration of resolution and artifact-free mosaic-stitching nature. Red and green colors denote third harmonic generation (THG) signal from hematoxylin and two-photon excitation fluorescence (TPEF) signal from eosin, respectively. Scale bar: 0.5 mm.

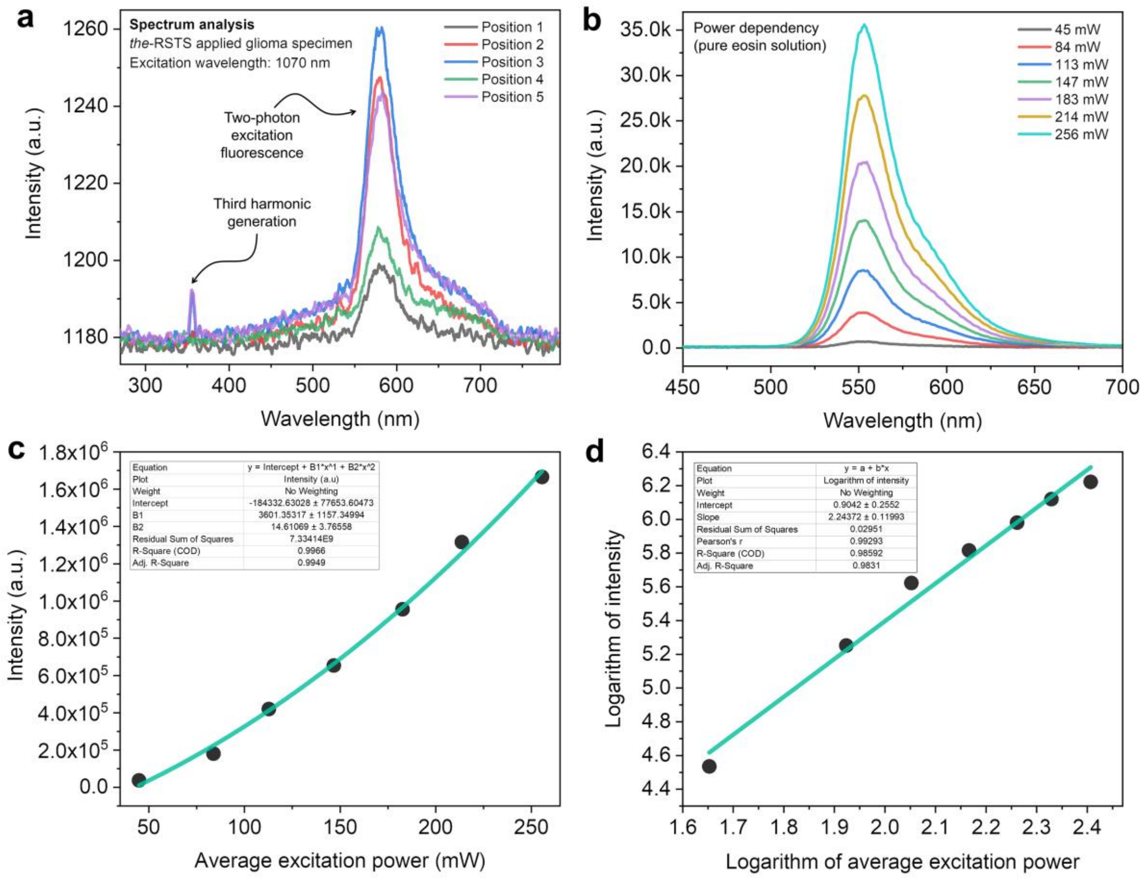

Supplementary Fig. 5 Spectroscopy analysis to investigate the sources of image contrast. **a** Spectrum detected from a glioma specimen applied with True-H&E Rapid whole-mount-Soft-Tissue Staining (the-RSTS) protocol. The first peak in (**a**) reveals third harmonic generation (THG) signal associated with hematoxylin-stained morphologies[4]. The second peak is contributed by two-photon excitation fluorescence (TPEF) signal from eosin dyes. **b** Spectrums from pure eosin solution recorded at varying average excitation power. **c** Plot of recorded signal intensity with respect to average excitation power. Data points are fitted with 2nd order polynomial. **d** Logarithmic plots for the data points in (**c**). Both (**c**) & (**d**) confirm quadratic power dependency of TPEF.

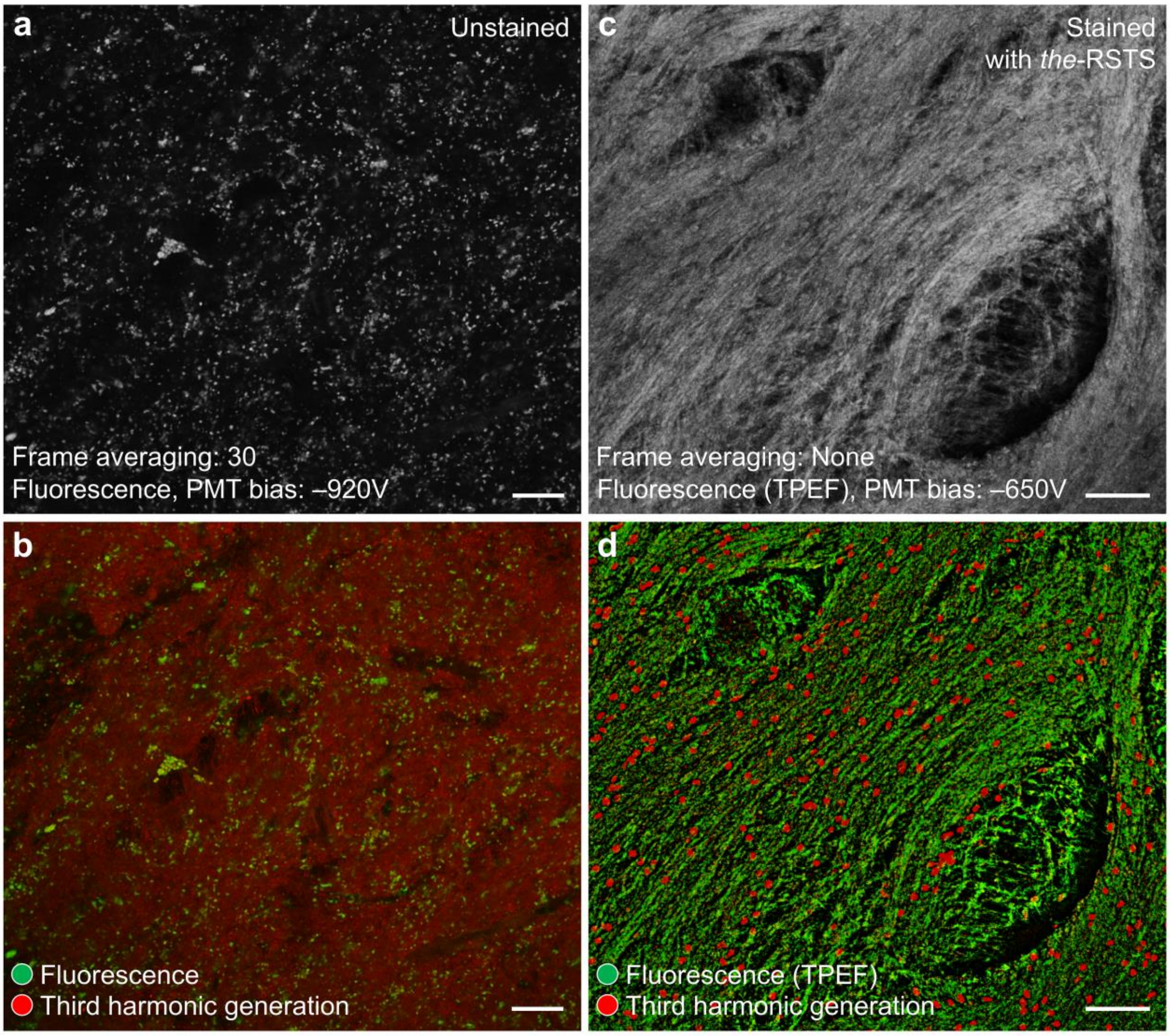

Supplementary Fig. 6 Analysis of fluorescence and third harmonic generation (THG) signals. **a** Autofluorescence signal detected at photomultiplier tube (PMT)-2 (filter: F2, 575/50, Edmund Optics; see Fig. 1a and *Methods*) from an unstained fresh human brain specimen at PMT biasing of –920V with averaging of 30 frames; **b** combined autofluorescence (green) and THG (red) signals for the unstained case. **c** Two-photon excitation fluorescence (TPEF) signal detected at the same channel (PMT 2 with F2) from the same specimen applied with our staining protocol, at PMT biasing of –650V with no frame averaging (single frame) at the same average excitation power; and **d** TPEF (green) and THG (red) signals merged for the stained case (applied with Denoised Contrast Enhancement). It is noted that for the stained case, we can easily see the TPEF signal (from eosin, see Supplementary Fig. 5) at PMT biasing of –650V. Signal strength was decent and no frame-accumulation was necessary. On the other hand, to see the autofluorescence signal for the unstained case, a much higher PMT biasing was required. Note that the imaging position before and after staining might not be the same owing to extra softness of the tissue. Scale bar: 50 µm.

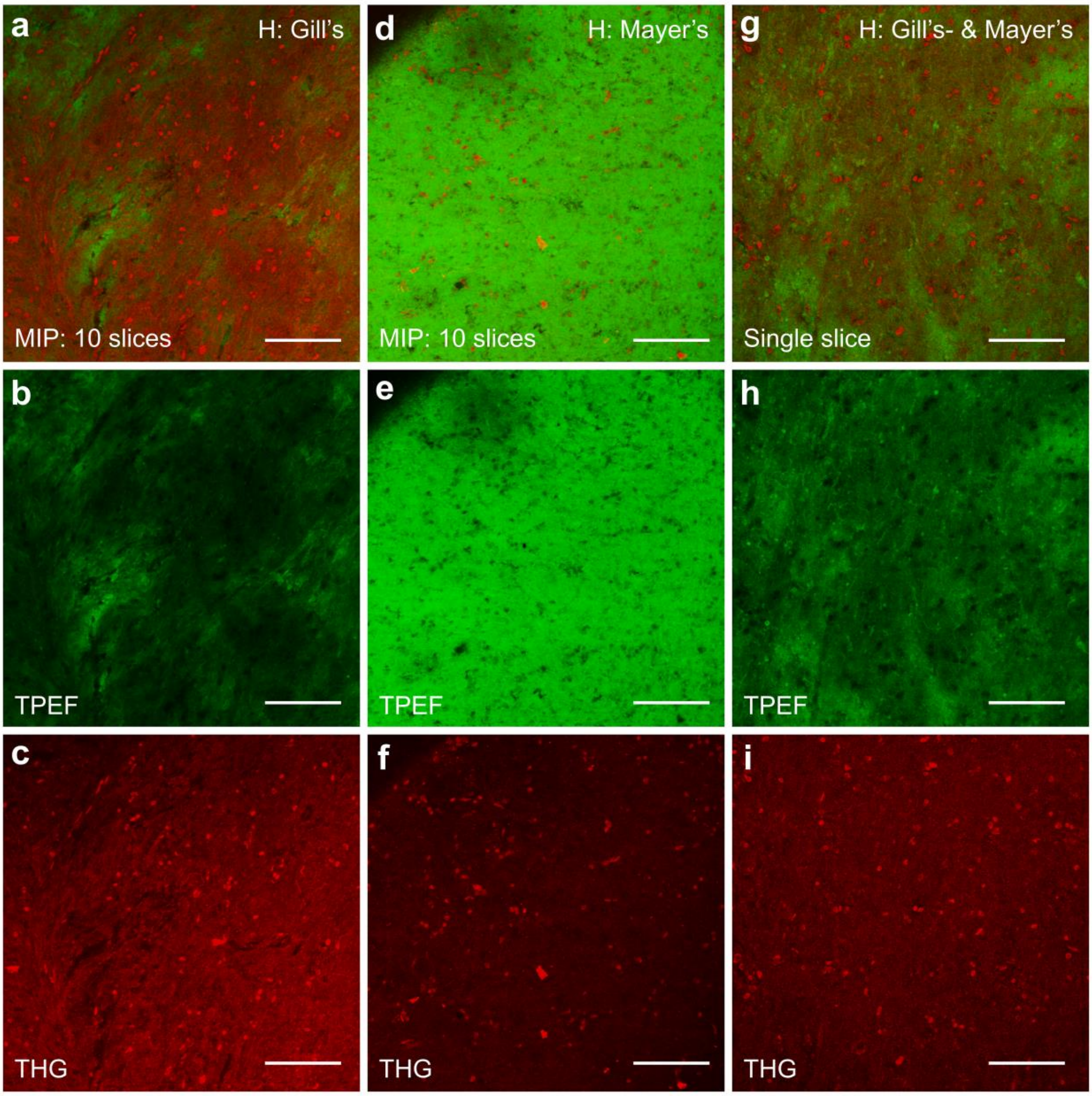

Supplementary Fig. 7 Experiments with Gill's & Mayer's hematoxylin solutions. **a**-**i** Cropped nonlinear optical images of a normal human brain specimen stained with (**a**-**c**) Gill's hematoxylin (H) followed by an eosin (E) staining, (**d**-**f**) Mayer's hematoxylin followed by an E-staining, and (**g**-**i**) both Gill's and Mayer's hematoxylin solutions followed by an E-staining (see our staining protocol in Supplementary Movie 1). Red and green channels in each case depict third harmonic generation (THG) and two-photon excitation fluorescence (TPEF) signals originating from the H and E dyes, respectively. Images in (**a**) & (**d**) were obtained with maximal intensity projection (MIP) of 10 slices collected at an axial step of 1 µm. In the case of Gill's hematoxylin, nuclei staining was promising, though the subsequent eosin staining quality was not that acceptable. On the other hand, in the case of Mayer's hematoxylin, the subsequent eosin staining was way stronger; however, the visibility of the cell nuclei became poor. The image in (**g**) is a single slice with no MIP performed. With combined Gill's & Mayer's hematoxylin solutions followed by E-staining, the overall image quality became promising. Scale bar: 100 µm.

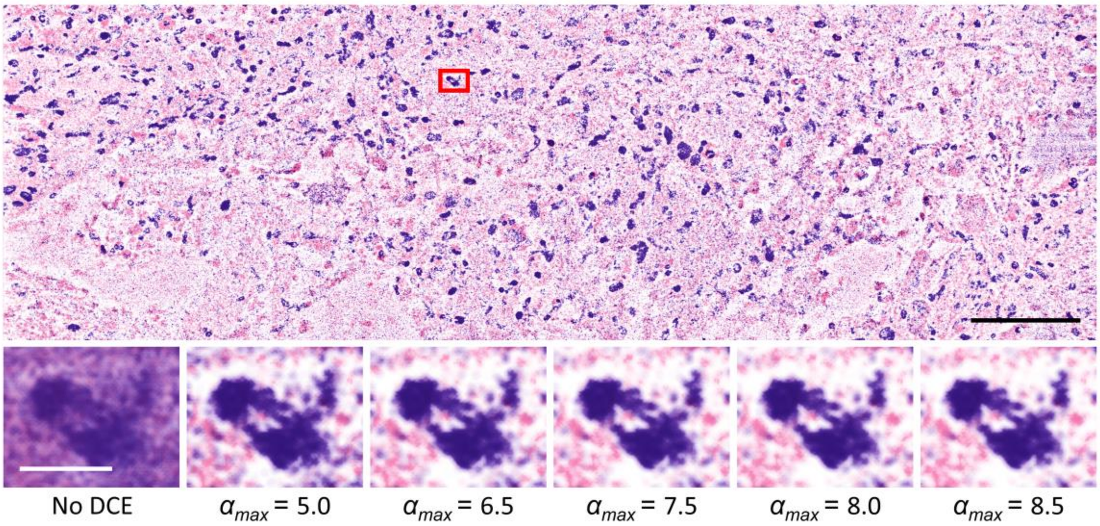

Supplementary Fig. 8 An example of mitosis observed in a True-H&E Rapid Fresh digital-Pathology (the-RFP) image. The red-marked region of interest (ROI) is cropped and enlarged for different conditions, i.e., with no Denoised Contrast Enhancement (DCE)[3] applied, and subsequently with enabled DCE for α_{max} values of 5.0, 6.5, 7.5, 8.0, and 8.5. It is noted that the chromatin in the cell was separated apart from one side to another. Scale bars for the original image and the cropped ROIs are 100 µm and 10 µm, respectively.

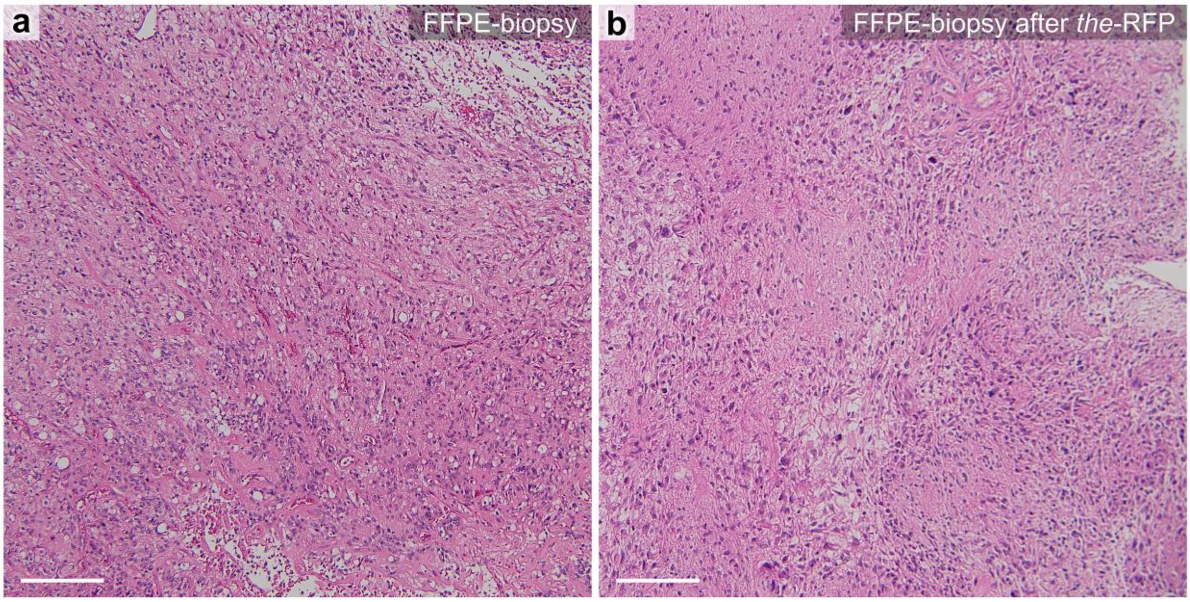

Supplementary Fig. 9 Transmission light microscopy images obtained by **a** directly performing a formalin-fixed paraffin-embedded (FFPE)-biopsy, and **b** first performing True-H&E Rapid Fresh digital-Pathology (the-RFP) procedure and subsequently an FFPE-biopsy. Each case utilizes an excised human brain tumor specimen. It is noted that a specimen processed with the-RFP can reliably undergo an FFPE-biopsy. Scale bar: 200 µm.

Supplementary Table 1 Estimation of compensating radial distortion parameters.

Tests	Vertically acquired tiles			Horizontally acquired tiles		
	A	X_{off}	Y_{off}	A	X_{off}	Y_{off}
1	25°	7	19	27°	5	17
2	25°	5	20	24°	6	20
3	25°	7	23	25°	6	21

Average values of A, X_{off}, and Y_{off} are found to be 25.2°, 6.0, and 20.0, respectively.

Supplementary Table 2 Steps involved in the sub-6-minute True-H&E Rapid whole-mount-Soft-Tissue Staining (the-RSTS) protocol (see Supplementary Movie 1).

	Used solutions	Time	Purpose
Step 1	10% neutral formalin	20 sec	Short Fixation
Step 2	Gill's hematoxylin	1 min 30 sec	Cell nucleus staining
Step 3	Mayer's hematoxylin	2 min 30 sec	Cell nucleus staining
Step 4	Distilled water	5 sec (twice)	Rinsing
Step 5	Ammonia	15 sec	Bluing
Step 6	Eosin Y (alcoholic)	15 sec	Staining of vessel, fiber, cell cytoplasm
Step 7	90% alcohol	10 sec	Rinsing

Supplementary references

1. Borah, B. J. *et al.* Nyquist-exceeding high voxel rate acquisition in mesoscopic multiphoton microscopy for full-field submicron resolution resolvability. *iScience* **24**, 103041 (2021).
2. Borah, B. J. & Sun, C.-K. Construction of a high-NFOM multiphoton microscope with large-angle resonant raster scanning. *STAR Protoc* **3**, 101330 (2022).
3. Borah, B. J. & Sun, C.-K. A rapid denoised contrast enhancement method digitally mimicking an adaptive illumination in submicron-resolution neuronal imaging. *iScience* **25**, 103773 (2022).
4. Sun, C. K. *et al.* Slide-free imaging of hematoxylin-eosin stained whole-mount tissues using combined third-harmonic generation and three-photon fluorescence microscopy. *J Biophotonics* **12**, e201800341 (2019).
