# Supplementary material for: Rapid digital pathology of H&E-stained fresh human brain specimens as an alternative to frozen biopsy
Source: Commun Med (Lond). 2023 May 30;3:77. doi: 10.1038/s43856-023-00305-w (PMC10229595; doi:10.1038/s43856-023-00305-w)
Supplement: Supplementary file 8 — Description of Additional Supplementary Files [file 43856_2023_305_MOESM8_ESM.pdf]

Description of Additional Supplementary File

File Name: Supplementary Movie 1

Description: Demonstration of the True-H&E Rapid whole-mount-Soft-Tissue Staining (the-RSTS) protocol.

File Name: Supplementary Movie 2

Description: Loading and visualization of a 1 Gigapixel custom bitmap.

File Name: Supplementary Movie 3

Description: Demonstration of the subminute gigapixel mesoscale Nonlinear Optical Gigascope (mNLOG) laser-raster-scanning platform.

File Name: Supplementary Movie 4

Description: Demonstration of the rapid artifact-compensated 2D large-field mosaic-stitching (rac2D-LMS) approach.

File Name: Supplementary Movie 5

Description: Demonstration of the True-H&E Rapid Fresh digital-Pathology (the-RFP) approach with a glioma specimen.

File Name: Supplementary Data 1
Description: The-RFP decisions and the respective comments from the pathologist in the non-interventional clinical study (diagnostic).
