# Supplementary material for: Rapid digital pathology of H&E-stained fresh human brain specimens as an alternative to frozen biopsy
Source: Commun Med (Lond). 2023 May 30;3:77. doi: 10.1038/s43856-023-00305-w (PMC10229595; doi:10.1038/s43856-023-00305-w)
Supplement: Supplementary file 9 — Reporting Summary [file 43856_2023_305_MOESM9_ESM.pdf]

nature portfolio

Corresponding author(s): Chi-Kuang Sun, Kuo-Chuan Wang

Last updated by author(s): May 10, 2023

Reporting Summary

Nature Portfolio wishes to improve the reproducibility of the work that we publish. This form provides structure for consistency and transparency in reporting. For further information on Nature Portfolio policies, see our Editorial Policies and the Editorial Policy Checklist.

Statistics

For all statistical analyses, confirm that the following items are present in the figure legend, table legend, main text, or Methods section.

n/a	Confirmed	
☐	☒	The exact sample size (*n*) for each experimental group/condition, given as a discrete number and unit of measurement
☐	☒	A statement on whether measurements were taken from distinct samples or whether the same sample was measured repeatedly
☒	☐	The statistical test(s) used AND whether they are one- or two-sided<br>*Only common tests should be described solely by name; describe more complex techniques in the Methods section.*
☒	☐	A description of all covariates tested
☒	☐	A description of any assumptions or corrections, such as tests of normality and adjustment for multiple comparisons
☒	☐	A full description of the statistical parameters including central tendency (e.g. means) or other basic estimates (e.g. regression coefficient) AND variation (e.g. standard deviation) or associated estimates of uncertainty (e.g. confidence intervals)
☒	☐	For null hypothesis testing, the test statistic (e.g. *F*, *t*, *r*) with confidence intervals, effect sizes, degrees of freedom and *P* value noted<br>*Give P values as exact values whenever suitable.*
☒	☐	For Bayesian analysis, information on the choice of priors and Markov chain Monte Carlo settings
☒	☐	For hierarchical and complex designs, identification of the appropriate level for tests and full reporting of outcomes
☒	☐	Estimates of effect sizes (e.g. Cohen's *d*, Pearson's *r*), indicating how they were calculated

Our web collection on statistics for biologists contains articles on many of the points above.

Software and code

Policy information about availability of computer code

Data collection	LASERaster+ v4.1.0.4 (Borah et al., 2022, 10.1016/j.xpro.2022.101330) was used for data acquisition and processing.
Data analysis	Compute Unified Device Architecture (CUDA, v11.4)-accelerated OpenCV (C++) v4.5.2 was used for data/image processing.

For manuscripts utilizing custom algorithms or software that are central to the research but not yet described in published literature, software must be made available to editors and reviewers. We strongly encourage code deposition in a community repository (e.g. GitHub). See the Nature Portfolio guidelines for submitting code & software for further information.

Data

Policy information about availability of data

All manuscripts must include a data availability statement. This statement should provide the following information, where applicable:

- Accession codes, unique identifiers, or web links for publicly available datasets
- A description of any restrictions on data availability
- For clinical datasets or third party data, please ensure that the statement adheres to our policy

The data/images generated and/or analyzed to support the findings of our study are presented in the paper and the Supplementary Information. More details would be available from the corresponding author upon reasonable request.

Human research participants

Policy information about studies involving human research participants and Sex and Gender in Research.

Reporting on sex and gender	Sex and gender were not relevant to this study.
Population characteristics	Tumor specific tissues were collected from subjects undergoing glioma resection surgeries with informed consent, and normal tissues were collected from preserved normal brain specimens (see Ethics oversight below).
Recruitment	Tissue collection was based on disease coverage and tissue availability from the Division of Neurosurgery, Department of Surgery, National Taiwan University Hospital; choices were random otherwise.
Ethics oversight	All experiments were performed as per the protocol reviewed and approved by the Research Ethics Committee of National Taiwan University and National Taiwan University Hospital. Institutional Review Board (IRB) approvals were taken under projects entitled “Microscopy Imaging of Hematoxylin-Eosin Stained Human Brain Tissues: Assessment margins of Excised Glioma Tumor in Surgery,” project number: 201912225RINB (National Taiwan University Hospital), and “Developing immunoassays for early detection of Alzheimer’s disease,” project number: 201412063RINC (National Taiwan University).

Note that full information on the approval of the study protocol must also be provided in the manuscript.

Field-specific reporting

Please select the one below that is the best fit for your research. If you are not sure, read the appropriate sections before making your selection.

☒ Life sciences ☐ Behavioural & social sciences ☐ Ecological, evolutionary & environmental sciences

For a reference copy of the document with all sections, see nature.com/documents/nr-reporting-summary-flat.pdf

Life sciences study design

All studies must disclose on these points even when the disclosure is negative.

Sample size	Based on a two-sided t-test (difference between two dependent means (matched pairs), G*Power v3.1.9.4), the total sample size was estimated to be at least 44 for a power of 90% at alpha level of 0.05 assuming a medium effect size of 0.5. We thus considered the-RFP datasets for 50 tissues with their respective FFPE-biopsy images for the blind test.
Data exclusions	No data were excluded.
Replication	All imaging attempts were successful.
Randomization	Tissue collection was based on disease coverage and tissue availability from the Division of Neurosurgery, Department of Surgery, National Taiwan University Hospital; choices were random otherwise. Both the-RFP and FFPE based datasets were assigned with randomized (hashed) IDs independently generated with hashing operations (prior to sending to the pathologists for assessment).
Blinding	Both the-RFP and FFPE based datasets were assigned with two sets of independently generated hashed IDs prior to sending to the respective pathologists. The hashing information was not known to any pathologist. Therefore, the pathologist viewing the-RFP datasets and the other pathologist viewing the FFPE datasets had no opportunity to correlate any case with each other. No communication between the pathologists occurred during the blind test.

Reporting for specific materials, systems and methods

We require information from authors about some types of materials, experimental systems and methods used in many studies. Here, indicate whether each material, system or method listed is relevant to your study. If you are not sure if a list item applies to your research, read the appropriate section before selecting a response.

Materials & experimental systems

n/a	Involved in the study
☒	☐ Antibodies
☒	☐ Eukaryotic cell lines
☒	☐ Palaeontology and archaeology
☒	☐ Animals and other organisms
☐	☒ Clinical data
☒	☐ Dual use research of concern

Methods

n/a	Involved in the study
☒	☐ ChIP-seq
☒	☐ Flow cytometry
☒	☐ MRI-based neuroimaging

Clinical data

Policy information about clinical studies

All manuscripts should comply with the ICMJE guidelines for publication of clinical research and a completed CONSORT checklist must be included with all submissions.

Clinical trial registration

N/A. The non-inferiority clinical study was diagnostic/non-interventional, with approved IRBs: 201912225RINB (National Taiwan University Hospital) and 201412063RINC (National Taiwan University).

Study protocol

The protocol is revealed in the manuscript, and additional information will be available from the corresponding author.

Data collection

Tumor-specific and normal brain samples (used for the blind non-inferiority clinical study; data collection time frame: October, 2021 to March, 2022) first underwent the-RSTS protocol, and the-RFP imaging was performed thereafter. For comparison purpose, FFPE biopsy was subsequently conducted and transmission light microscopy images were collected for each case.

Outcomes

The-RFP based tumor-vs-benign binary decision for each case matched the respective FFPE-biopsy outcome.
